# Evaluation of the relationship between the 14-3-3ε protein and LvRab11 in the shrimp *Litopenaeus vannamei* during WSSV infection

**DOI:** 10.1038/s41598-021-97828-w

**Published:** 2021-09-28

**Authors:** Guson Boonyoung, Tanate Panrat, Amornrat Phongdara, Warapond Wanna

**Affiliations:** 1 Division of Biological Science, Faculty of Science, Prince of Songkla University, Hat Yai, Songkhla, 90110 Thailand; 2 Center for Genomics and Bioinformatics Research, Prince of Songkla University, Hat Yai, Songkhla, 90110 Thailand; 3 Prince of Songkla University International College, Hat Yai Campus, Prince of Songkla University, Hat Yai, Songkhla, 90110 Thailand

**Keywords:** Biotechnology, Molecular biology

## Abstract

The 14-3-3 proteins interact with a wide variety of cellular proteins for many diverse functions in biological processes. In this study, a yeast two-hybrid assay revealed that two 14-3-3ε isoforms (14-3-3ES and 14-3-3EL) interacted with Rab11 in the white shrimp *Litopenaeus vannamei* (LvRab11). The interaction of 14-3-3ε and LvRab11 was confirmed by a GST pull-down assay. The *LvRab11* open reading frame was 645 bp long, encoding a protein of 214 amino acids. Possible complexes of 14-3-3ε isoforms and LvRab11 were elucidated by in silico analysis, in which LvRab11 showed a better binding energy score with 14-3-3EL than with 14-3-3ES. In shrimp challenged with the white spot syndrome virus (WSSV), the mRNA expression levels of *LvRab11* and *14-3-3ε* were significantly upregulated at 48 h after challenge. To determine whether LvRab11 and binding between 14-3-3ε and LvRab11 are active against WSSV infection, an in vivo neutralization assay and RNA interference were performed. The results of in vivo neutralization showed that LvRab11 and complexes of 14-3-3ε/LvRab11 delayed mortality in shrimp challenged with WSSV. Interestingly, in the RNAi experiments, the silencing effect of *LvRab11* in WSSV-infected shrimp resulted in decreased *ie-1* mRNA expression and WSSV copy number. Whereas suppression of complex *14-3-3ε/LvRab11* increased WSSV replication. This study has suggested two functions of LvRab11 in shrimp innate immunity; (1) at the early stage of WSSV infection, LvRab11 might play an important role in WSSV infection processes and (2) at the late stage of infection, the 14-3-3ε/LvRab11 interaction acquires functions that are involved in immune response against WSSV invasion.

## Introduction

The 14-3-3 protein family is highly conserved and expressed in all eukaryotic organisms. 14-3-3 proteins are known for their ability to bind multiple cellular proteins^1,2^. More than one hundred 14-3-3 binding partners are involved in cell cycle regulation, apoptosis, signal transduction, protein trafficking and stress responses^3^. A number of 14-3-3 isoforms are found in various organisms. In shrimp, two 14-3-3 epsilon (14-3-3ε) isoforms, 14-3-3ES and 14-3-3EL have been reported in *Litopenaeus vannamei*^4^. The expression of *14-3-3ε* mRNA increases significantly after white spot syndrome virus (WSSV) infection. However, the function of 14-3-3 in shrimp is still unclear. In *Drosophila melanogaster*, 14-3-3ε is required for the Rab11-positive vesicle function, which in turn enables antimicrobial peptide secretion during an innate immune response^5^. 14-3-3ε mutants were present in accumulations of small Rab11-positive vesicles near the plasma membrane, but RNAi silencing of *Rab11* significantly blocked the anterograde delivery of an antimicrobial peptide (drosomycin) from the perinuclear region to the plasma membrane.

Rab proteins constitute the largest group of the Ras superfamily of small G-proteins found to be present in all eukaryotes^6^. Of the large number of Rab proteins, 11 isoforms are known to be involved in the immune response endocytic pathway, namely, Rab3a, Rab4a, Rab4b, Rab5a, Rab5b, Rab5c, Rab7a, Rab9a, Rab9b, Rab11a and Rab11b^7^*.* Rab11 mediates several cellular processes that involve intracellular vesicle trafficking, including the delivery of plasma membrane proteins to specialized sites and the secretion of various factors. Rab11-mediated trafficking events were reported to have a role in several aspects of innate immunity^8^, in *Macrobrachium rosenbergii*, MrRab11A was involved in antibacterial and anti-WSSV innate immunity in prawns^9^. Moreover, in *Penaeus monodon*, PmRab11 was required for yellow head virus (YHV) infection^10^.

In the preliminary investigation of the function of 14-3-3ε in shrimp, a yeast two-hybrid assay was used to identify the binding ligands of the isoform, and Rab11 was found to be one of the binding partners. Therefore, this research focused on determining the relationship between 14-3-3ε and LvRab11 in *L. vannamei* using in vivo, in vitro and in silico analyses. In addition, molecular biological experiments were used to detail the characteristics and innate immune response function of LvRab11 in *L. vannamei* during viral infection.

## Results

### LvRab11 is a 14-3-3ε-interacting protein

**Figure 1:**
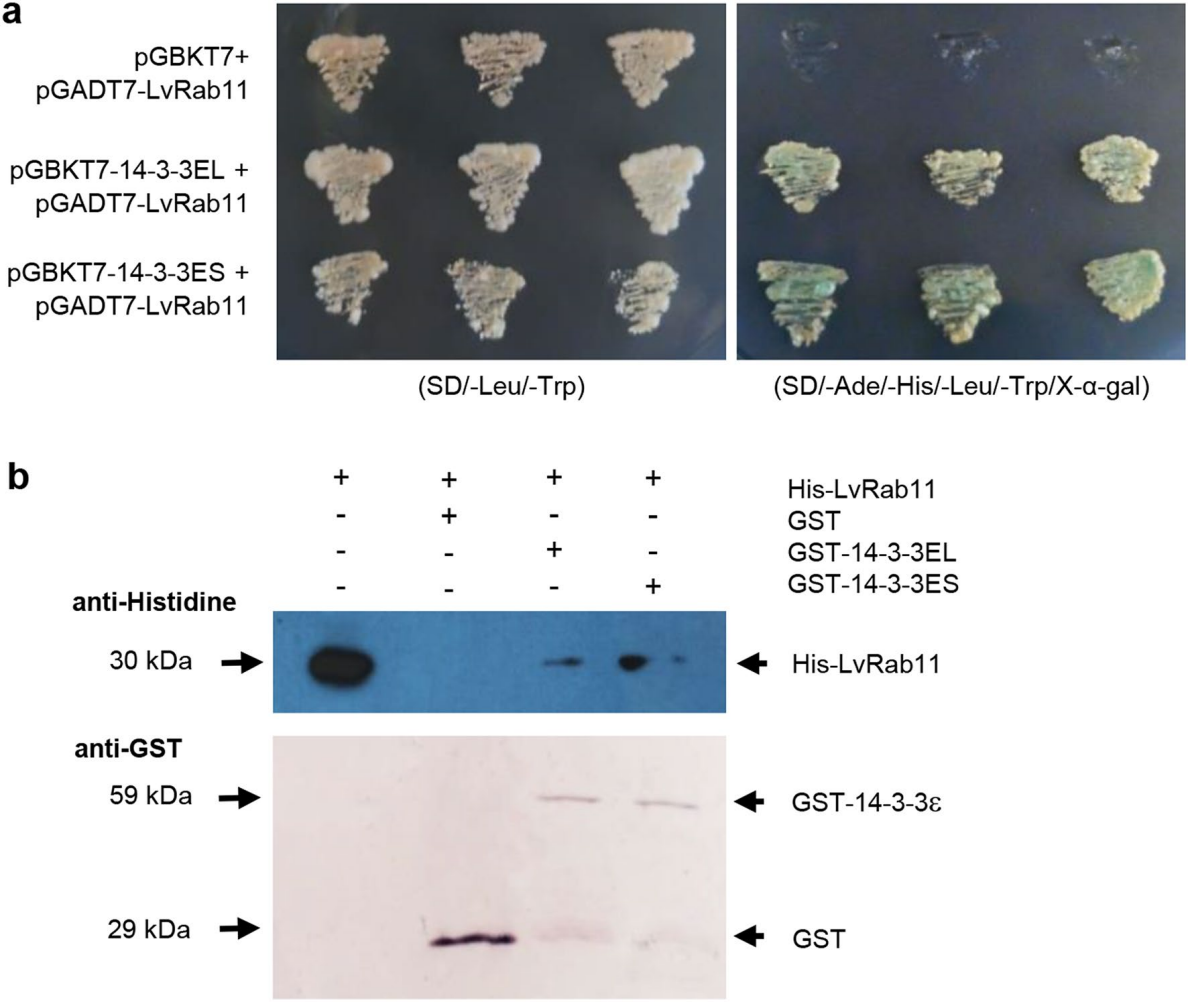
Evaluation of the 14-3-3ε and LvRab11 interaction. (**a**) Interaction was demonstrated by a yeast two-hybrid assay. AD-LvRab11 and BD-14-3-3ES or BD-14-3-3EL were co-transformed into the AH109 strain. Yeast cells with the two plasmids were cultured in SD-Leu/-Trp medium, selecting for the presence of both plasmids, and in SD-Leu/-Trp/-His/-Ade/X-α-gal medium, in which only the yeast cells with protein interactions were capable of growth and showed blue colonies. (**b**) Interaction was demonstrated by GST pull-down assays. GST pull-down assays using purified GST-14-3-3ES and GST-14-3-3EL recombinant proteins detected an interaction with His-LvRab11. The His-LvRab11 proteins in binding reactions were detected with a goat anti-mouse horseradish peroxidase-conjugated antibody bound to a mouse anti-histidine antibody (top panel). The GST, GST-14-3-3ES and GST-14-3-3EL proteins in binding reactions were detected with a rabbit anti-goat alkaline phosphatase-conjugated antibody bound to a goat anti-GST antibody (bottom panel).

A yeast two-hybrid assay was conducted to screen proteins from the shrimp hemocytes cDNA library that interact with 14-3-3ε. The prey plasmids were isolated and subjected to DNA analysis, which identified sequences of *Rab11*. To determine the interaction, BD-14-3-3ES, BD-14-3-3EL and AD-LvRab11 were cotransformed into yeast cells. BD-14-3-3ES, BD-14-3-3EL and AD-LvRab11 protein interactions manifested as blue colonies on a high-stringency medium containing X-α-Gal, while a lack of interaction led to no growth (Fig. 1a).

We confirmed the binding of LvRab11 to 14-3-3ε with in vitro GST pull-down assays, performed using purified GST-14-3-3ES, GST-14-3-3EL and His-LvRab11 recombinant proteins. The results showed that His-LvRab11 binds to GST-14-3-3ES and GST-14-3-3EL but does not bind to GST (Fig. 1b). In addition, we used the database of the Eukaryotic Linear Motif (ELM) resource (http://elm.eu.org/searchdb.html) to detect functional sites of LvRab11. The search revealed a 14-3-3-binding phosphopeptide motif (129-RHLRSVP-135) in the LvRab11 protein (Supporting Fig. 1). This bioinformatics result supported the idea that LvRab11 may interact with both 14-3-3ES and 14-3-3EL isoforms.

**Figure 2:**
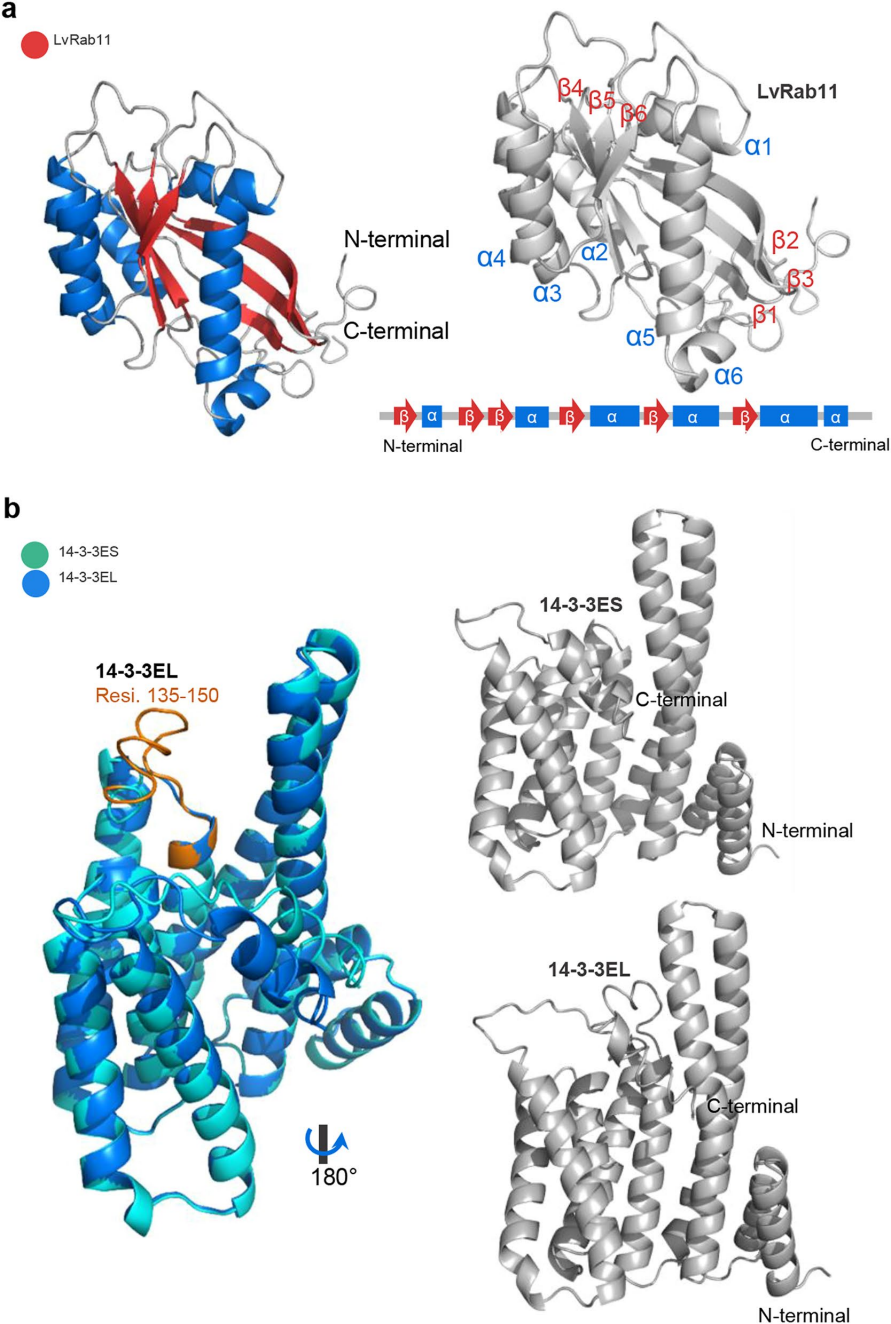
Predicted 3D structure of 14-3-3 and LvRab11. A 3D structure model of hub binding proteins was generated from the primary amino acid sequence by using homology modelling on the SWISS-Model server^11–13^ combined with threading-based LOMETS on the I-TASSER server^14–16^ and the pDomTHREADER search method on the PSIPRED server^17,18^. (**a**) The cartoon structure presenting the predicted model of LvRab11. (**b**) Predicted structure of 14-3-3 isoforms, where 14-3-3EL is coloured blue-marine and 14-3-3ES is coloured turquoise. The insertion residues of 14-3-3ε found at amino acid residues 135–150 in the 14-3-3EL are coloured in the orange fragment.

To investigate the details of the 14-3-3ε/LvRab11 interaction complexes, we first created 3D models of LvRab11 and the 14-3-3ES and 14-3-3EL isoforms derived from amino acid sequences. We used the online SWISS-Model server, the threading-based LOMETS resource on the I-TASSER server and the pDomTHREADER searching method on the PSIPRED server. In the predicted 3D model of LvRab11 and 14-3-3ε, 14-3-3ES is coloured green-turquoise and 14-3-3EL is blue-marine (Fig. 2). The predicted structure of LvRab11 shows six α-helices and six β-sheets (Fig. 2a). The extended region of the 14-3-3EL isoform is represented in orange in the amino acid residues Thr135–Glu150, which are located in the flexile region between the 5th α-helix (amino acid residues Ala111–Leu134) and the 6th α-helix (amino acid residues Phe151–Pro181) at the centre of the 14-3-3EL structure (Fig. 2b). The quality of the final prediction models of LvRab11 and the 14-3-3ES and 14-3-3EL isoforms is shown in Supporting Figs. 2–4.

**Figure 3:**
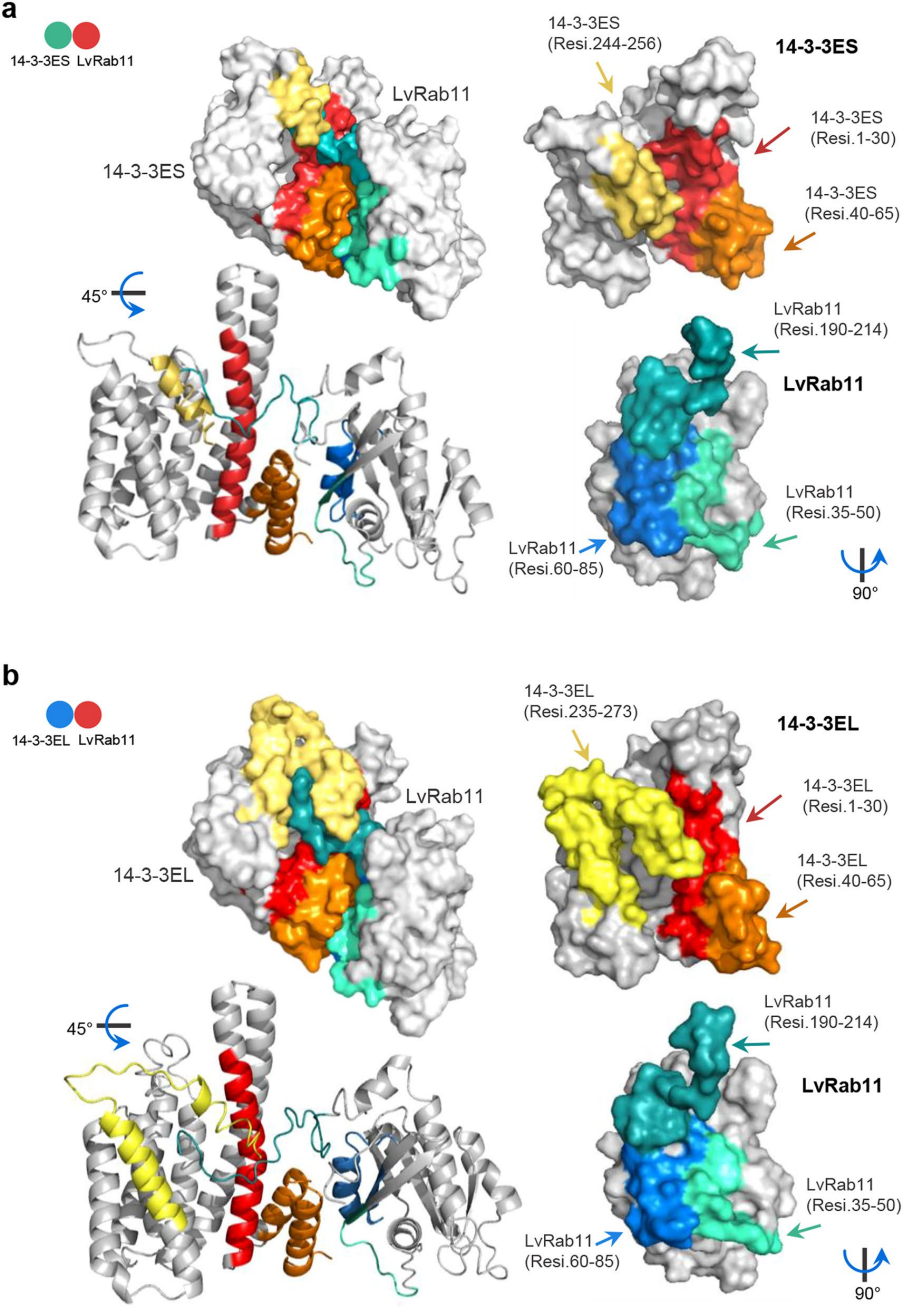
Docking simulation of 14-3-3 isoforms and the LvRab11 Interaction complex. 14-3-3 and LvRab11 were docked using the default parameters of the ClusPro 2.0 server^19,20^ and AutoDock software^21,22^. The first ranking of the simulated complex was selected to explore the binding sites in detail. (**a**) We assigned a 3D model of 14-3-3ES (coloured turquoise) as a receptor and LvRab11 (cherry-red) as a ligand for protein interaction complex simulation. (**b**) Interaction complex of 14-3-3EL:LvRab11, where 14-3-3EL is coloured blue-marine and LvRab11 is coloured cherry-red. The interaction structures of 14-3-3ε and LvRab11 were generated using PyMOL molecular graphic version 2.4.1 (https://pymol.org).

To analyse the possible complex structure and lock-and-key binding features of the LvRab11 protein and 14-3-3ε isoforms, we used the ClusPro 2.0 online docking servers and AutoDock Vina to simulate the interaction complexes that may promote the activation of innate immune responses to viral infection. Two simulation approaches were used in this in silico study: a one-on-one simulation and a competitive complex simulation. In the one-on-one simulation, we assigned the LvRab11 protein as the ligand molecule and the 14-3-3ε (14-3-3ES and 14-3-3EL) protein as the receptor molecule. The one-on-one simulation results found that the LvRab11/14-3-3EL complex shows a higher binding energy score of interaction than LvRab11/14-3-3ES, with the centre binding energy and the lowest binding energy being − 1000.60 kcal/mol and − 1021.10 kcal/mol, respectively, whereas the values for LvRab11/14-3-3ES were − 751.00 kcal/mol and − 867.70 kcal/mol, respectively (Table 1). Moreover, the predicted structural analysis of the interaction complex found that three amino acid regions may act as binding sites of LvRab11/14-3-3ε, whereas the amino acid residues of 14-3-3ES at Met1-Val30, Glu40-Ser65, and Gln244-Ser256 (Fig. 3a) and those of 14-3-3EL at Met1-Val30, Glu40-Ser65, and Leu235-Ser273 (Fig. 3b) bound to LvRab11 at Glu35-Thr50, Ile60-Val85, and Pro190-Arg214, respectively.

**Table 1:** Docking﻿ simulation results for 14-3-3ε and LvRab11.

Interaction partners and Binding sites		Binding energy (Kcal/mol)	
Receptor	Ligand	Center	Lowest
**14-3-3 and LvRab11**			
**14-3**-**3ES**	**LvRab11**	− 751.00	− 867.70
1**–**30, 40**–**65, 244**–**256	35**–**50, 60**–**85, 190**–**214		
**14**-**3**-**3EL**	**LvRab11**	− 1000.60	− 1021.10
1**–**30, 40**–**65, 235**–**273	35**–**50, 60**–**85, 190**–**214		
**14**-**3-3 Dimerization simulation**			
**14**-**3-3ES**	**14-3-3ES**	− 754.90	− 754.90
1–30, 55–95, 250–257	1–30, 55–95, 250–257		
**14-3-3EL**	**14-3-3EL**	− 700.50	− 716.60
1–30, 55–95, 267–273	1–30, 55–95, 267–273		
**14-3-3EL**	**14-3-3ES**	− 788.80	− 793.80
1-30, 55-95, 267-273	1-30, 55-95, 250-257

**Figure 4:**
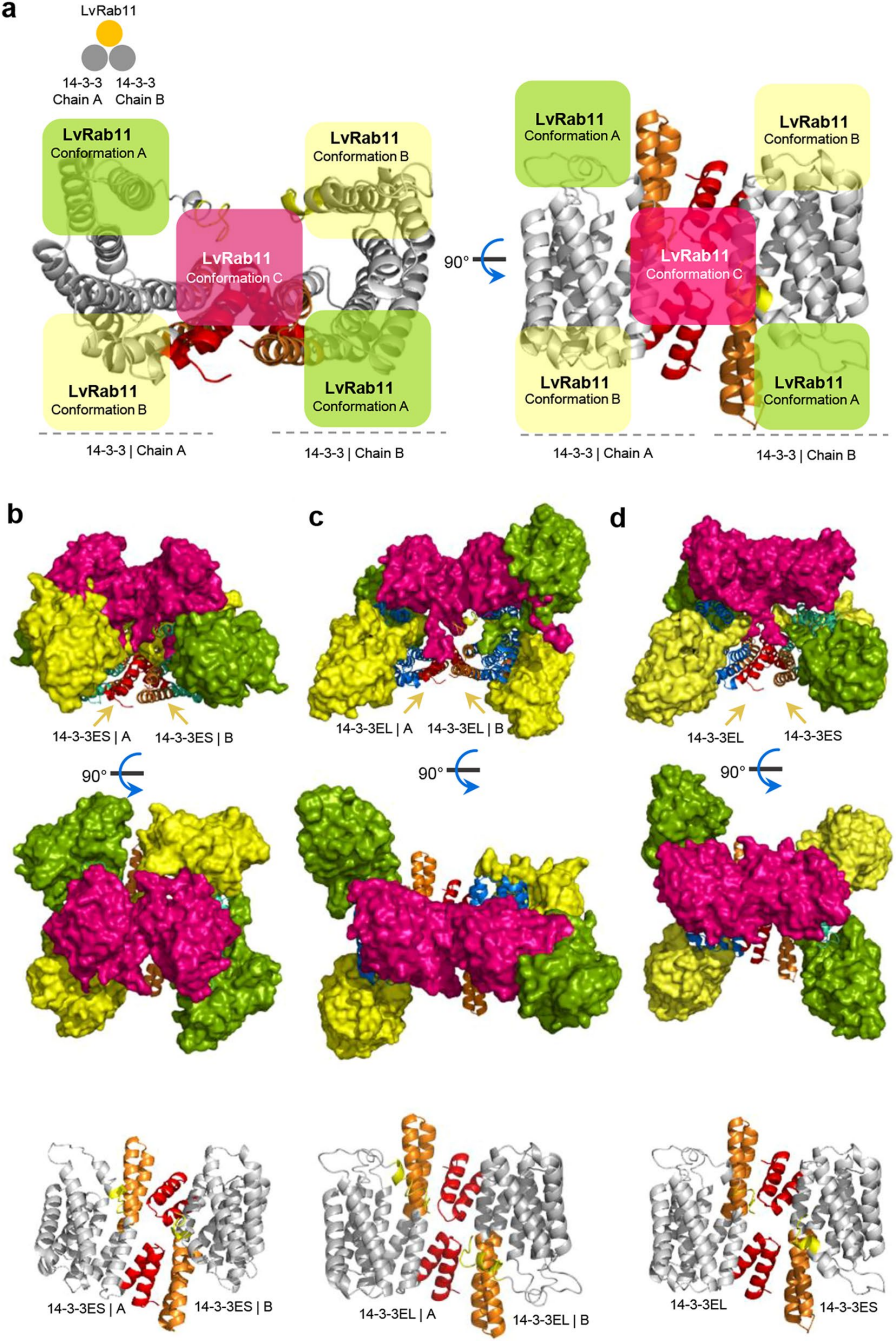
Competitive simulation of 14-3-3ε dimer complexes with LvRab11 as a receptor protein. The structure represents the competitive structures of the 14-3-3ε dimer interacting with LvRab11. (**a**) The symbols show the simulated model of LvRab11 as a receptor interacting with 14-3-3ε isoforms as the ligand proteins. The modelled LvRab11 structure is coloured chalky-pink, and the 14-3-3ε structures are in green-lemon and golden-yellow. (**b**-**d**) The illustration shows the competitive structures of the possible conformation of the 14-3-3ε dimer interacting with LvRab11. The PyMOL version 2.4.1 (https://pymol.org) was applied for rendering the competitive complexes of 14-3-3ε dimers interacting with LvRab11.

To investigate the competitive binding between the 14-3-3ε dimer complexes and the LvRab11 protein, we assigned the 14-3-3ε structured dimer as a receptor and LvRab11 as a ligand. We obtained three successful combinations of the 14-3-3 dimers and LvRab11 protein: (1) 14-3-3ES/ES:LvRab11, (2) 14-3-3EL/EL:LvRab11, and (3) 14-3-3EL/ES:LvRab11 (Fig. 4a). The analysis of the 14-3-3ES/ES:LvRab11 competitive structure (Fig. 4b) found that the 14-3-3ES/ES:LvRab11 structure gave the best binding score, with a centre binding energy of -804.40 kcal/mol and lowest binding energy of − 804.40 kcal/mol. The possible region that might assist 14-3-3 molecules in binding with residues Met1–Ser40 and Gln180–Arg214 of the LvRab11 structure is located at amino acids Ala195-Asp250 near the C-terminus. Whereas the 14-3-3EL/EL:LvRab11 competitive structure (Fig. 4c) analysis found the best binding score for the 14-3-3EL/EL:LvRab11 structure, with a centre binding energy of − 874.50 kcal/mol and lowest binding energy of − 856.80 kcal/mol. Two binding regions of the 14-3-3EL/EL dimer are located at residues Ala175-Asp223 and Leu225-Ser273, and the binding regions of the LvRab11 structure are located at Met1-Ser20, and Ile175-Arg214. The simulated competitive structure of 14-3-3EL/ES:LvRab11 (Fig. 4d) has the best centre binding energy (− 839.20 kcal/mol) and the best lowest binding energy (− 839.20 kcal/mol). The LvRab11 binding interaction sites were present at amino acids Met1-Ser25, Ser40-Val85, and Gln180-Arg214. The 14-3-3EL/ES dimer structure shows a variety of interaction regions, whereas the 14-3-3EL molecule shows the possible residues at Val35-Ser65, Gly102-Phe120, Lys169-Ile185, and Leu225-Ser273, and 14-3-3ES shows the possible residues at Val31-Ala55, Asp105-Phe120, Ala155-Leu175, and Ala195-Ser257. The binding energy results for the competitive docking simulation and analysis results are shown in Table 2 and Fig. 4.

**﻿Table 2:** Competitive binding simulation results for 14-3-3ε complexes and LvRab11.

Interaction partners, Binding sites			Possible conformation	Average score of binding energy (Kcal/mol)	
Receptor (14-3-3 Dimer)		Ligand (LvRab11)		Center	Lowest
**14-3-3ES/ES**		**LvRab11**			
70–80, 130–245		35–50, 60–85, 100–120, 185–214	A	− 778.05	− 822.75
195–250		1–40, 180–214	B	− 804.40	− 804.40
1–45, 200–257		1–55, 190–214	C	− 754.35	− 844.85
**14-3-3EL/EL**		**LvRab11**			
131–160, 195–205, 245–273		1–15, 175–214	A	− 745.40	− 780.55
175–273		1–20, 175–214	B	− 874.50	− 856.80
1–45, 220–273		25–55, 70–80, 175–214	C	− 738.15	− 804.60
**14-3-3EL/ES**		**LvRab11**			
**14-3-3EL**	**14-3-3ES**				
65–85, 190–210, 240–255	55–155, 230–257	1–20, 40–50, 65–85, 175–214	A	− 735.20	− 826.10
35–65, 102–120, 169–185, 225–273	31–55, 105–120, 155–175, 195–257	1–25, 40–85, 180–214	B	− 839.20	− 839.20
225–273	200–257	1–50, 60–85, 180–214	C	− 743.30	− 776.45

### Identification of *LvRab11* in* L. vannamei*

In our previous study, using a yeast two-hybrid assay, 14-3-3ε was shown to interact with a partial Rab11 sequence from a cDNA library. However, to confirm this interaction in *Litopenaeus vannamei,* the full-length cDNA of *Rab11* of *L. vannamei* (*LvRab11*) was identified and characterized (GenBank accession no. MW429225). The sequence of *LvRab11* cDNA was 645 bp long, encoding 214 amino acids. The amino acid sequence of LvRab11 shared the highest similarity (99%, 96% and 83% identities) with Rab11 from *P. monodon* (GenBank accession no. ASW35116.1), *M. rosenbergii* (GenBank accession no. NP_173136) and *D. melanogaster* (GenBank accession no. BAA21708), respectively. Alignment of the LvRab11 protein and other Rab11 proteins indicated that LvRab11 possesses many structural features of the Rab11 subfamily. LvRab11 contained five highly conserved GTP-binding sites, five Rab family motifs, four Rab subfamily motifs (RabSF1-RabSF4), and a Cys–Cys motif at the carboxyl-terminus (Supporting Fig. 1). LvRab11 was identified as a Rab11a homologue sharing amino acid sequence similarity with Rab11-like proteins from other eukaryotes.

To evaluate the evolutionary relationships of LvRab11 with Rab11 proteins in other organisms, a phylogenetic tree was constructed using the neighbour-joining method. LvRab11 was clustered with the crustacean proteins PmRab11 from *P. monodon* and MrRab11A from *M. rosenbergii* and was in the invertebrate group (Supporting Fig. 5).

**Figure 5:**
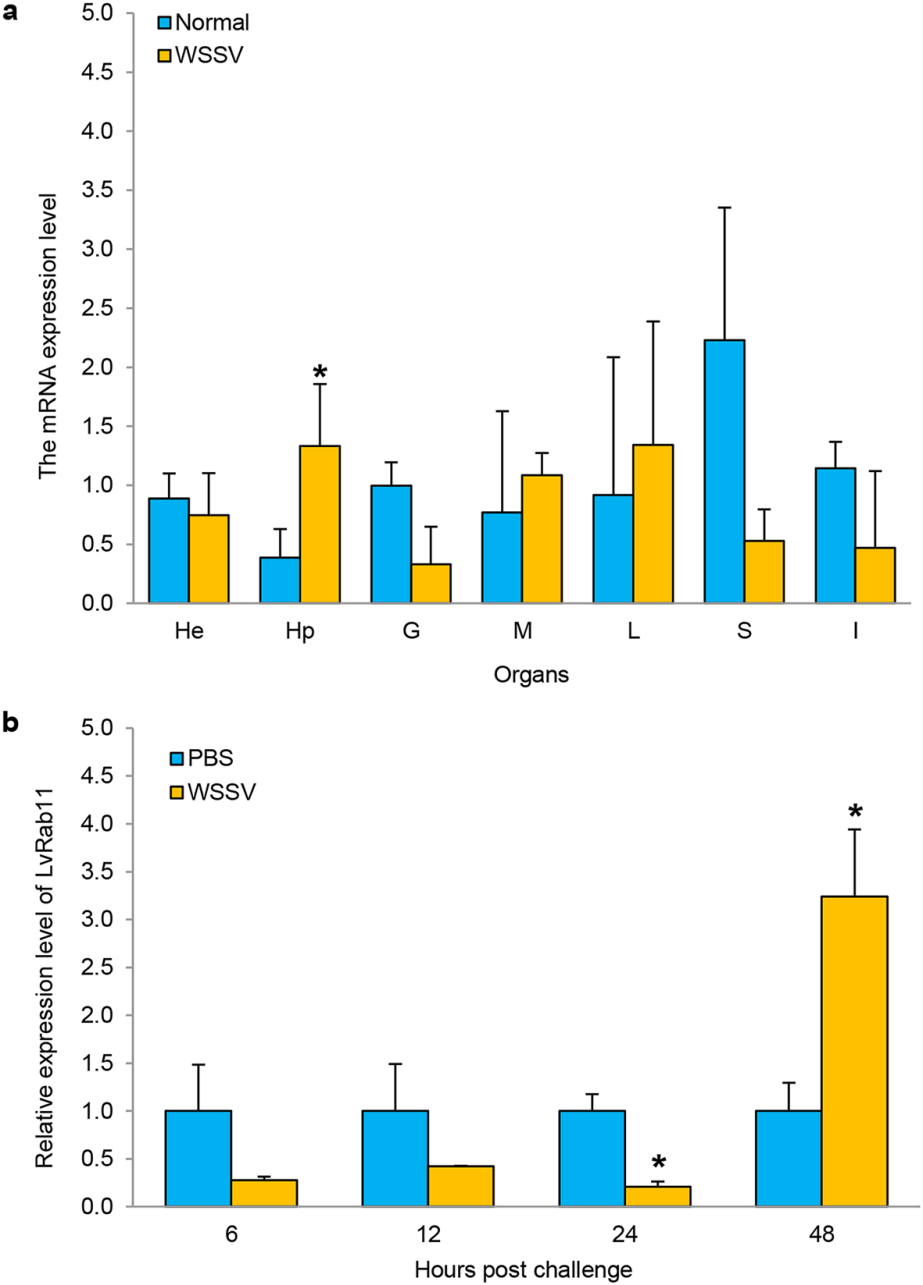
Expression profile of the *LvRab11* transcripts in *L. vannamei.* (**a**) Gene expression of *LvRab11* in various tissues of shrimp challenged with WSSV at 48 h post injection. The tissues examined included hemocytes (He), hepatopancreas (Hp), gill (G), muscle (M), lymphoid (L), stomach (S) and intestine (I), n = 3/tissue. (**b**) The expression profiles of *LvRab11* were analysed in the hepatopancreas of WSSV-challenged shrimp at various time points during infection, n = 3/time point. Error bars indicate standard deviations. Asterisks indicate significant differences (*p* < 0.05) compared with the values of the control.

### Expression analysis of the *LvRab11* and *14-3-3ε* in shrimp

**Figure 6:**
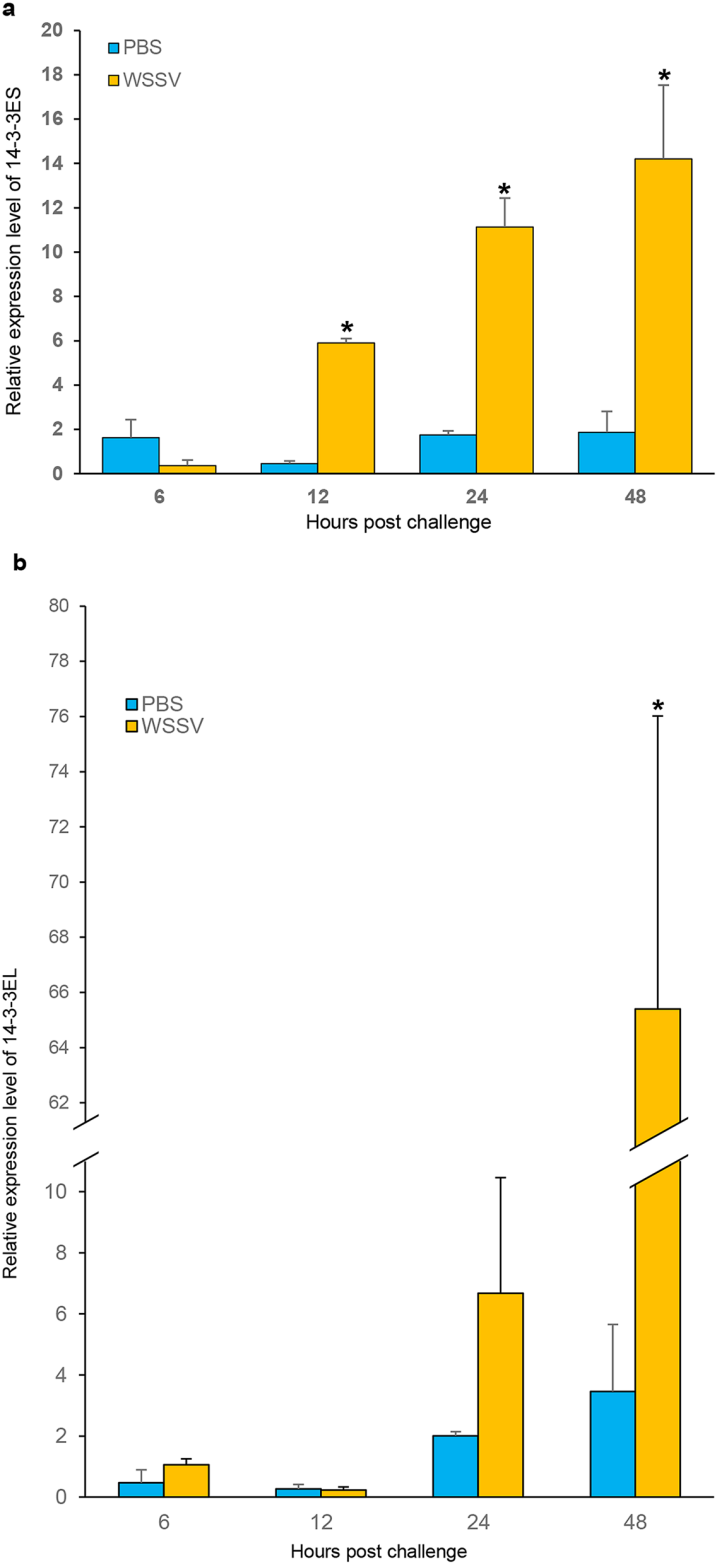
Expression profile of the *14-3-3ε* transcripts in *L. vannamei* at various time points during WSSV infection*.* The expression profiles of *14-3-3ES* (**a**) and* 14-3-3EL* (**b**) were determined in the hepatopancreas of WSSV- challenged shrimp at various time points during infection. Error bars indicate standard deviations (n = 3/time point). Asterisks indicate significant differences (*p* < 0.05) compared with the values of the control.

The mRNA expression of *LvRab11* in various tissues was detected by RT-PCR. *LvRab11* transcripts were expressed in all the tissue samples analysed (Supporting Fig. 6). The expression level of *LvRab11* in various tissues of shrimp upon challenge with WSSV was determined using RT-PCR. The results revealed that *LvRab11* mRNA was significantly upregulated in the hepatopancreas after WSSV infection (Fig. 5a). We also quantified the transcripts of *LvRab11* and *14-3-3ε* in the hepatopancreas of WSSV-infected shrimp at various time points. The results showed that the *LvRab11* and *14-3-3EL* mRNA levels were significantly increased at 48 h post-infection (hpi) (Figs. 5b, 6b). Whereas *14-3-3ES* transcripts were upregulated from 12 to 48 hpi when compared to PBS-injected group (Fig. 6a). Upregulation of *LvRab11* and *14-3-3ε* transcript levels implies that these genes might play an important role in immune response after WSSV infection.

### In vivo neutralization

**Figure 7:**
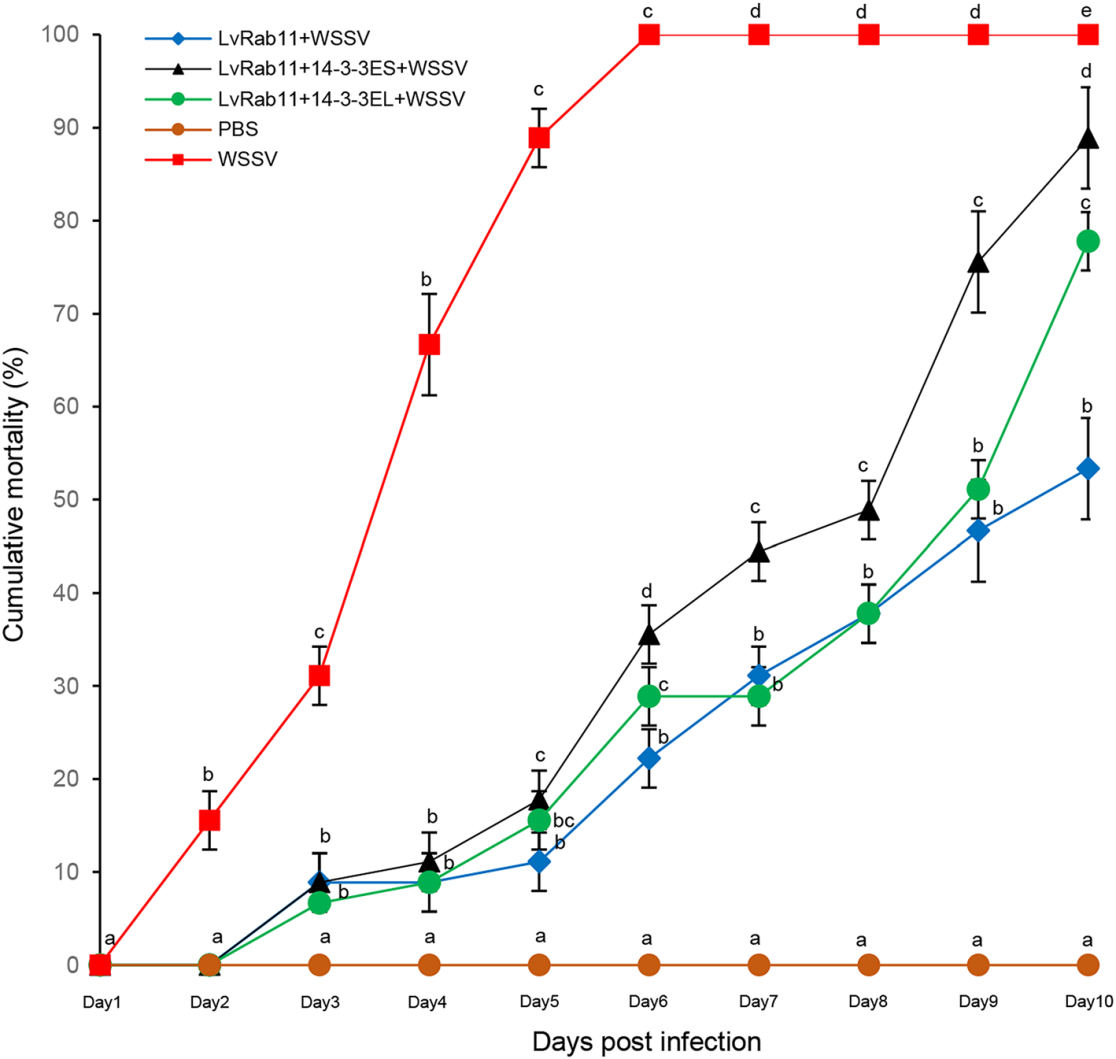
Cumulative post challenge shrimp mortality. The solutions used for shrimp injection are shown on the left. Cumulative mortality data represent the pooled results for 3 replications (n = 15 for each group). Error bars indicate standard deviations. Different letters indicate statistically significant difference between five groups (*p* < 0.05).

An in vivo neutralization assay was set up to determine whether LvRab11 and 14-3-3ε are involved in WSSV infection in shrimp. There was no shrimp mortality in the PBS-infected group, but shrimp infected with WSSV alone showed an increasing mortality as the test progressed and 100% mortality was reached at day 6. However, low mortality was observed in the three remaining groups at 6 days post injection. For LvRab11 group, mortality was 22% at day 6 and reached 53% at day10. For the complex of LvRab11/14-3-3ES, mortality was 36% at day 6 and 89% at day 10. The complex of LvRab11/14-3-3EL, 29% mortality was observed at day 6 and 78% mortality at day10 (Fig. 7). The experiments were done twice, and similar results were observed. Thus, the results indicated that LvRab11 alone, and the complexes of 14-3-3ε/LvRab11 can delay WSSV infection or block white spot syndrome in shrimp.

### Silencing efficiency of LvRab11 and 14-3-3ε dsRNA in shrimp during WSSV infection

**Figure 8:**
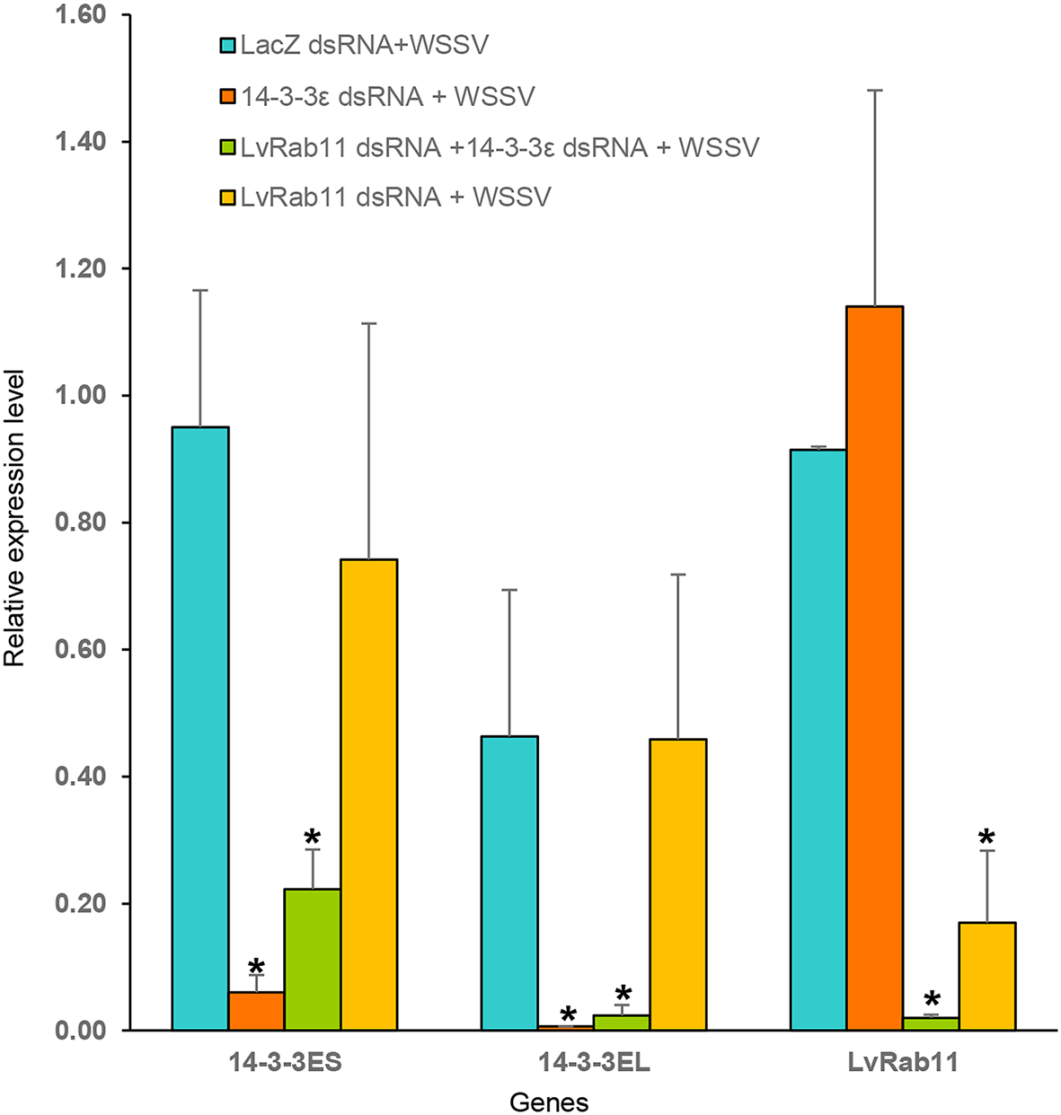
Suppression of *LvRab11* and *14-3-3ε* in shrimp during WSSV infection. Expression level of *LvRab11* and *14-3-3ε* was detected from the gill of the shrimps injected with 20 μg of each dsRNA following WSSV infection. The data represent means ± standard deviations, n = 3/group. Asterisks indicate significant differences (*p* < 0.05) compared with the values for the control.

To investigate whether *LvRab11* and *14-3-3ε* mRNA expressions can be suppressed by using the RNAi approach. The dsRNAs targeting *LvRab11* and *14-3-3ε* were produced and injected into shrimp muscle. The comparison of *LvRab11* and *14-3-3ε* mRNA level in shrimp from 4 groups including (1) LacZ dsRNA + WSSV, (2)14-3-3ε dsRNA + WSSV, (3) LvRab11 dsRNA + WSSV, and (4) LvRab11 and 14-3-3ε dsRNA + WSSV is shown in Fig. 8. The results showed that the transcription level of *LvRab11* in* LvRab11*-silenced group and in both knockdown groups were significantly lower than that in shrimp from the control LacZ dsRNA + WSSV group, and the 14-3-3ε dsRNA + WSSV group. Using 14-3-3ε dsRNA, the results demonstrated that the expression of *14-3-3ES* and *14-3-3EL* transcripts were significantly decreased in the 14-3-3ε dsRNA + WSSV group and in both LvRab11 and *14-3-3ε* silenced groups. However, the expression of the *LvRab11* transcript was not affected by *14-3-3ε* knockdown.

### Effects of *LvRab11* and *14-3-3ε* dsRNA gene silencing in WSSV-challenged shrimp

**Figure 9:**
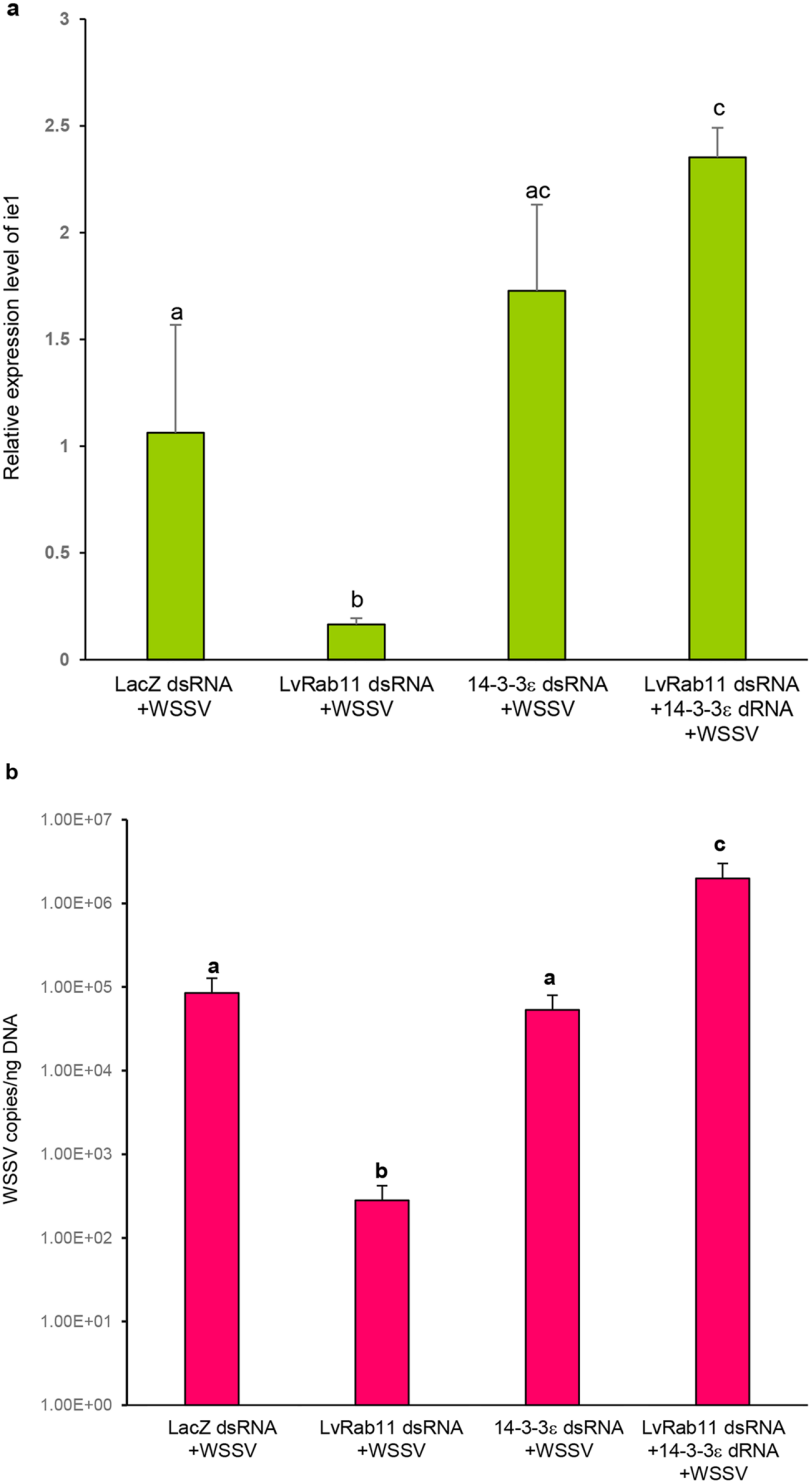
Effect of *LvRab11* and *14-3-3ε* dsRNA gene silencing in WSSV-challenged shrimp. (**a**) Relative expression of *ie1* transcript was detected in shrimp at 48 h after WSSV infection. (**b**) Suppression effect on WSSV copy number was monitored in shrimp challenged with WSSV. The data represent means ± standard deviations, n = 3/group. Different letters indicate statistically significant difference between four groups (*p* < 0.05).

The roles of LvRab11 and 14-3-3ε during WSSV infection were investigated by RNA interference. The results showed that *ie1* mRNA expression level and WSSV copy number in *LvRab11* knockdown group were significantly decreased after 48 h post WSSV infection compared to the other groups. This contrasts with the result of both the knockdown groups (*LvRab11* + *14-3-3ε*) where *ie1* transcript and WSSV level were significantly increased after WSSV infection when compared to the control group and the *LvRab11* knockdown group. However, the *14-3-3ε* knockdown groups revealed that *ie1* mRNA and WSSV level were significantly increased when compared to the *LvRab11* knockdown group but not significant when compared to the control group (Fig. 9).

## Discussion

The proteins of the 14-3-3 family play critical roles in a wide range of cellular functions through interactions with client proteins. Their crucial role in health and disease is suggested by the association of 14-3-3 proteins with the regulation of a wide range of general and specific signalling pathways^23^. Furthermore, 14-3-3 family members play a significant role in interactions with several RNA and DNA viral proteins through multiple pathways^24^. They also interact with client proteins and influence their activity, localization, stability, or protein–protein interactions (PPIs) and, consequently, have an effect on viral infection ^24–27^.

In our previous study, 14-3-3ε formed both heterodimers and homodimers. The expression profiles of the two 14-3-3ε isoforms were altered after viral infection, indicating that the shrimp 14-3-3ε isoform is probably related to cellular processes that are often modulated during viral infection in *L. vannamei* ^4^. However, the function of 14-3-3ε that is required depends on the type of cell in which 14-3-3 is present. In this study, to understand the role of the 14-3-3ε isoforms, the binding partners during virus infection were investigated. The results obtained from the yeast two-hybrid (in vivo) and GST pull-down (in vitro) assay revealed that LvRab11 interacted with both 14-3-3ε isoforms. Two high-affinity phosphorylation-dependent 14-3-3 binding motifs, RSXpSXP (mode 1) and RXXXpSXP (mode 2), have been reported^28,29^. Analysis of the LvRab11 amino acid sequence revealed a 14-3-3-binding phosphopeptide motif (129-RHLRSVP-135) belonging to mode 2. These results imply that the 14-3-3ε isoform can bind both phosphorylated and nonphosphorylated LvRab11. Although most 14-3-3 proteins have been shown to interact with phosphoserine-containing peptides^30^, there are also several reports showing an interaction between 14-3-3 proteins and nonphosphorylated ligands^31,32^. There are structural similarities between the phosphorylated and nonphosphorylated ligands.

In the possible complexes of 14-3-3ε isoforms and LvRab11 obtained from our in silico analysis, LvRab11 molecules occupied most of the central space in the channel of the 14-3-3ε dimers. However the binding energy score of LvRab11 was better with 14-3-3EL than with 14-3-3ES. The simulated complexes suggest that amino acid Met1–Val30 at the N-terminus of the 14-3-3 structure is the recognition region for self-interaction at amino acids Ala55–Asp95. Moreover, the amino acid residues 54–GARRASWRIISSIEQKE–70 of 14-3-3ES and 14-3-3EL also present a pseudosubstrate domain. The serine residue at the C-terminus (Ser257 of 14-3-3ES and Ser273 of 14-3-3EL) may facilitate the formation of an active and inactive structure of 14-3-3ε for binding with its binding partner. Both the N-terminus and C-terminus of LvRab11 displayed regions that may play key roles in interacting with the 14-3-3 protein. Near the C-terminus of the 14-3-3ε isoform, the EF-hand motif domain with the pattern “LSEESYKDSTLI” (at Leu209-Ile220 of 14-3-3ES and Leu225-Ile236 of 14-3-3EL) forms a helix-loop-helix topology and may support the interaction of the 14-3-3 protein with ligand molecules within the HF-loop. The helix-loop-helix topology strongly supports the crystal structure of the 14-3-3ε protein, which exhibits a highly helical and cup-shaped dimer^33^.

*LvRab11* was identified from the hemocytes of *L. vannamei*. Sequence analysis revealed that the LvRab11 protein contains five GTP-binding sites (G1-G5). The Ras superfamily has the ability to bind GTP^34^, and Rabs are distinguished from other members of the Ras GTPase family by five Rab family motifs (RabF1-5) and four Rab subfamily motifs (RabSF1-4)^35^. Moreover, the LvRab11 protein exhibited a prenylation site (CC) at its C-terminus; prenylation is a crucial post-translational modification that enables Rab proteins to associate with and target cell membranes^35^. Based on homology searches in the GenBank database, the LvRab11 protein was highly homologous with Rab11 of other species and had the closest phylogenetic relationship with PmRab11^10^ and MrRab11A^9^*.* Thus, the LvRab11 protein might be closely associated with biological functions in cellular processes in shrimp. *LvRab11* expression was observed in all the tissues tested in the shrimp but at different levels. Hepatopancreas tissue is important for the production of immune response molecules, it does not only initiate humoral immune, but also play an important role in cellular immune response^36,37^. In hepatopancreas, *LvRab11* and 14-3-3ε expression profile were significantly increased in the late stage of WSSV stimulation (48 hpi), therefore, we imply that LvRab11 and 14-3-3ε represents a supplemental immune molecule that functions in the late stage of WSSV infection. Several studies have shown that the expression level of 14-3-3 isoform was upregulated after virus infection, and overexpression of 14-3-3 protein could reduce the production of virus particles^4,38,39^. Santos et al.^36^ reported that genes upregulated in hepatopancreas and in healthy shrimp (shrimps exposed to the virus but not infected) are likely to be involved in response to WSSV and are central ones in keeping shrimps WSSV free. Whereas downregulation of *LvRab11* in early stage in this tissue may imply that the virus may inhibit this gene to favor its replication in the initial stage^40^.

We also found that LvRab11 alone and the complex of LvRab11 and 14-3-3ε reduced shrimp mortality through neutralizing the WSSV infection. In agreement with in silico data, this results of an in vivo neutralization showed that complex of LvRab11 and 14-3-3EL delayed shrimp mortality more than complex of LvRab11 and 14-3-3ES. Several studies have confirmed that Rab proteins play a significant role in the immune system^37,41–44^. In addition, many Rab proteins play critical roles in the assembly and passage of enveloped viruses from cells^45^. In humans, Rab11 proteins have been implicated in the replication of many viral pathogens. For example, respiratory syncytial virus, cytomegalovirus and human immunodeficiency virus require Rab11 and its adapter to assemble in host cells^45^. Several viruses also use Rab11-dependent pathways for transport^46–50^.

In our RNAi experiment, silencing *LvRab11* by RNAi resulted in a decreased *ie1* mRNA expression and WSSV replication. The *ie1* is an early-immediate transcription gene that is highly expressed throughout the WSSV infection cycle. It plays an important role in promoting viral infection and replication^51^. Down-regulation of *ie1* gene and WSSV level after LvRab11 silencing may indicate the possible role of LvRab11 proteins in intracellular trafficking of WSSV in the early stage of infection. Similar results were reported in PmRab11 and PmRab7. Silencing *PmRab11*^10^, *PmRab7*^52,53^ or *LvRab7*^54^ resulted in inhibition of viral replication in shrimp. For instance, the co-localization between PmRab11 and YHV was observed in hemocytes at 24 to 72 h post viral injection, whereas in *PmRab11* knockdown hemocytes, a low level of co-localization of PmRab11 and YHV was detected at the perinuclear region at 24–72 h. These results revealed that PmRab11 is required for YHV infection in shrimp cells^10^. In addition, silencing of *PmRab7* resulted in significant inhibition of WSSV-VP28 mRNA and YHV replication, suggesting that PmRab7 required for WSSV and YHV infection in shrimp cell^52^.

The silencing *14-3-3ε* alone resulted in increased *ie1* mRNA expression and WSSV level in shrimp compared to LvRab11 knockdown group but not significant when compared to the control group. However, when knockdown of both *LvRab11* and *14-3-3ε* were performed, the result showed that *ie1* transcript and WSSV replication were significantly increased when compared to *LvRab11* knockdown and control groups. In the same way, with in vivo neutralization, Rab11 and the complex of Rab11 and 14-3-3ε can delay mortality of shrimp after WSSV infection. The result of which may imply that LvRab11 might also have a role in the shrimp defense mechanism when binding to 14-3-3ε in the late stage of infection. After a virus is infected into the host cell cytoplasm, viral molecules may trigger binding of 14-3-3ε especially 14-3-3EL to LvRab11. Then, the LvRab11/14-3-3ε interaction may activate LvRab11 to turn on the specific function to protect the cell against WSSV infection. In most cases, 14-3-3 proteins bind onto target proteins at the original site, and upon certain stimulations, the 14-3-3 proteins will then bring their target proteins to specific locations where the proteins can properly functions^55–57^. Some Rabs such as Rab6 have been shown to play important roles in WSSV infection. It plays a critical role in the regulation of phagocytosis against WSSV by the rearranging the actin fibers^58,59^. In addition, suppression of *Rab6* by specific dsRNA has significantly increased WSSV copy number in shrimp^60,61^.

Hence, we can hypothesize that LvRab11 may have two functions during WSSV infection; (1) In the early stage, LvRab11 plays a role in the endocytic process and it is required for WSSV infection and (2) In the late stage, the interaction between 14-3-3ε and LvRab11, 14-3-3ε may be an activating binding partner LvRab11 protein and contribute an immune response against WSSV invasion.

However, this is the first report of the interaction between 14-3-3ε and LvRab11 in shrimp. In Drosophila, 14-3-3ε is required for Rab11-positive vesicle function, which in turn enables antimicrobial peptide secretion during the innate immune response^5^. There are some reports about Rab proteins interacting with 14-3-3, such as 14-3-3 being an interaction partner for AS160 (containing a Rab GTPase-activating domain), indicating an important role for this protein in insulin-regulated GLUT4 trafficking^62,63^.

In conclusion, knowledge of the viral infection mechanism and the associated cellular response is very important and provides insight into host–pathogen interactions that can be used to develop an improved approach for viral infection prevention in shrimp. Although we found an interaction between 14-3-3ε and LvRab11 in this study that may be related to an immune response after viral infection, further elucidation of the cellular mechanisms is needed to better understand the role of these interactions during viral infection and pathogenesis in host cells.

## Materials and methods

### Yeast two-hybrid assay

The recombinant plasmids pGBKT7-14-3-3ES and pGBKT7-14-3-3EL were obtained from previous studies. Here, a cDNA library from hemocytes of WSSV-infected *P. monodon*, that was constructed in pGADT7 and used to screen for interaction, was introduced into *Saccharomyces cerevisiae* strain AH109 using the Matchmaker GAL4 Two-Hybrid System 3 (Clontech Laboratories, Inc.). Transformed yeast cells were spread on synthetic dropout selection medium such as low-stringency (SD/-leucine/-tryptophan), medium-stringency (SD/-leucine/-tryptophan/-adenine) or high-stringency (SD/-adenine/-histidine/-leucine/-tryptophan) medium at 30 °C. The interaction produced blue colonies in a high-stringency medium containing X-α-Gal. The cDNA library plasmids from interaction-positive colonies were sent for DNA sequencing. The full-length *LvRab11* was cloned into pGADT7 (pGADT7-LvRab11) to confirm its interaction with pGBKT7-14-3-3ES and pGBKT7-14-3-3EL. The cotransformation containing pGBKT7-53 and pGADT7-T was used as the positive control, and that of pGBKT7 with pGADT7- LvRab11 or pGADT7 was employed as the negative control.

### Identification of *LvRab11* and sequence analysis

A partial fragment of *LvRab11* from *L. vannamei* was amplified using degenerate primers designed according to the conserved sequences of *Rab11* genes such as the *Homo sapiens* (GenBank accession no. X56740), *Mus musculus* (GenBank accession no. AB232605), *D. melanogaster* (GenBank accession no. D84315) and *Danio rerio* (GenBank accession no. NM_001007359) in the GenBank database (http://www.ncbi.nlm.nih.gov). *LvRab11* was amplified by PCR using the Rab11-F and Rab11-R primers. The PCR cycles were conducted as follows: 95 °C for 5 min, followed by 35 cycles of 95 °C for 1 min, 52 °C for 1 min, and 72 °C for 1 min and 72 °C for 10 min. The PCR product was purified and ligated into the pGEM-T easy vector (Promega) and sequenced.

To obtain the full-length *LvRab11* gene, the open reading frame (ORF) of *LvRab11* was amplified with LvRab11-specific primers (fullLvRab11-F and fullLvRab11-R) using the PCR conditions were as described above. Sequences were analysed with the BLASTX program (https://blast.ncbi.nlm.nih.gov/Blast.cgi). The LvRab11 protein motifs were analysed by InterPro (https://www.ebi.ac.uk/interpro/search/sequence-search). All primer sequences are presented in the supporting information (Supporting Table 1).

### Construction of a phylogenetic tree

Multiple sequence alignment of Rab11 was performed with the ClustalX program. The amino acid sequences of Rab11 were used to construct phylogenetic trees by the neighbour-joining method using MEGAX software. The aligned sequences were bootstrapped 1,000 times to test the relative support for a particular clade.

### Analysis of *LvRab11* mRNA expression in shrimp tissues

Healthy *L. vannamei* specimens (body weight 7–10 g) were obtained from Songkhla, Thailand. Various tissues, including the hemocytes, hepatopancreas, gill, intestine, lymphoid, muscle and stomach, were collected for RNA extraction with TRIzol reagent (Invitrogen). First-strand cDNA was synthesized from 1 µg of total RNA using the SuperScript III First-Strand Synthesis System kit (Invitrogen) according to the manufacturer’s protocols. The tissue distribution of *LvRab11* in normal shrimp and in WSSV-infected shrimp (48 hpi) were analysed by semi-quantitative PCR using the primers Rab11-RT-F and Rab11-RT-R. The PCR conditions were as described above. The *β-actin* gene was amplified as an internal standard. The band intensity of *LvRab11* and *β-actin* in gel was measured by densitometry through ImageJ software 1.37v (National Institutes of Health, Bethesda USA). The mRNA expression levels of *LvRab11* were normalized against those of *β-actin*.

### Expression pattern of the *LvRab11* and *14-3-3ε* genes in WSSV-infected shrimp

The shrimp were divided into the control and WSSV-challenged groups. The shrimp in the WSSV-challenged group were injected with a 100 µl suspension of WSSV (3.9 × 10^3^ copies). In the control group, 100 µl of PBS were injected into each shrimp. The expression pattern of the *LvRab11* and *14-3-3ε* genes in the hepatopancreas of WSSV-infected shrimp was investigated at 6, 12, 24, and 48 h to study the role of the gene in the innate immunity of *L. vannamei*. Reverse transcription quantitative real-time PCR (RT-qPCR) was performed using the CFX Connect Real-Time PCR Detection System (Bio-Rad Laboratories) with SensiFAST SYBR No-ROX Mix (Bioline). The thermal cycling profile used was 95 °C for 3 min, followed by 30 cycles of 95 °C for 30 s, 52 °C for 30 s (for *LvRab11*) or 60 °C for 30 s (for *14-3-3ES* and *14-3-3EL*) and 72 °C for 30 s. An additional temperature ramping step from 55 °C to 95 °C was utilized to produce melting curves for the reaction. *EF-1α* gene was amplified as an internal standard, and relative fold changes were calculated using the the formula 2^−ΔΔCT^ method.

### Plasmid construction, protein expression and purification

*LvRab11*, *14-3-3ES* and *14-3-3EL* were cloned into the pET-28a (+) vector (Novagen) that already contained an N-terminal 6xHis tag. The protein was expressed in *Escherichia coli* strain BL21 (DE3) and purified using HisPur Ni–NTA Superflow Agarose (Thermo Scientific) following the manufacturer’s instructions. GST-fusion 14-3-3ε proteins were subcloned into the pGEX-4T-1 vector (Amersham Biosciences). The recombinant GST-14-3-3EL and GST-14-3-3ES proteins were produced in *E. coli* strain BL21 and purified using Glutathione Sepharose 4 Fast Flow (GE Healthcare) following the manufacturer’s instructions. Purified recombinant proteins were analysed by SDS-PAGE. The protein concentration was measured with a dye-binding assay by the Bradford method and stored at − 20 °C.

### Glutathione S-Transferase (GST) pull-down assay and western blot analysis

In the pull-down experiments, 30 µg of GST and the GST-14-3-3ES and GST-14-3-3EL fusion proteins were bound to glutathione Sepharose beads and washed five times with 1x PBS. Thirty micrograms of 6xHis-LvRab11 were incubated with GST or one of the GST-fusion proteins for 1 h. The complexed beads were washed ten times using 1x PBS. Finally, 5 × SDS sample loading buffer was added to the complexed beads, and the samples were boiled for 5 min.

The proteins were separated by SDS-PAGE, and proteins were transferred to nitrocellulose membranes and blocked with 5% skim milk. The proteins were detected using the GST tag as follows. The membrane was incubated with primary antibody (goat anti-GST, 1:5,000) for 1 h, washed three times with 1x PBS, incubated with alkaline phosphatase-conjugated secondary antibody (rabbit anti-goat alkaline phosphatase (AP), 1:10,000) for 1 h, and washed three times with 1x PBS. The substrate (NBT/BCIP) was added to the membrane and the proteins were visualized by color detection.

Western blotting was performed according to standard procedures. The proteins were detected by using the His tag as follows. The membrane was incubated with mouse anti-His primary antibody (1:2,000) and goat anti-mouse horseradish peroxidase (HRP) secondary antibody (1:5,000). The ECL substrate was added to the membrane and the protein bands were visualized by exposing X-ray film to the membrane.

### Rab11/14-3-3ε isoform complex simulation and analysis

Freely available online tools, namely, the SWISS-Model server (https://swissmodel.expasy.org)^11–13^, the threading-based LOMETS resource on the I-TASSER server (https://zhanglab.ccmb.med.umich.edu/I-TASSER)^14–16^, and the pDomTHREADER search method on the PSIPRED server (http://bioinf.cs.ucl.ac.uk/psipred)^17,18^, were used for predicting the three-dimensional (3D) structures of the 14-3-3ε isoforms and the Rab11 protein. The online docking servers ClusPro 2.0 (https://cluspro.bu.edu)^19,20^, AutoDock suite (available for free download at http://autodock.scripps.edu)^21^ and AutoDock Vina (available for free download at http://vina.scripps.edu)^22^ were used for the lock-and-key model analysis of the interaction sites of the 14-3-3 isoforms and the Rab11 protein. The PyMol molecular viewer was used to visualize and analyse the details of the 3D structure models^64^.

### In vivo neutralization assay

His-14-3-3ES, His-14-3-3EL and His-LvRab11 were expressed and purified in order to use in the in vivo neutralization assay. Before starting the experiments, the *L. vannamei* specimens (approximately 3 g) were acclimated for at least 3–5 days. They were then divided into 5 groups (15 shrimps per group) for hemocoel injection with (1) 1x PBS as negative control, (2) WSSV (10^5^ copies/shrimp) as positive control, (3) LvRab11 (5 µg/shrimp) plus WSSV, (4) LvRab11 (3 µg/shrimp) and 14-3-3EL (3 µg/shrimp) plus WSSV, and (5) LvRab11 (3 µg/shrimp) and 14-3-3ES (3 µg/shrimp) plus WSSV. Each group was done in triplicates. The experiments were carried twice. The shrimps were kept under standard conditions and mortality was recorded daily for 10 days.

### dsRNA production and RNAi performance

Sense and anti-sense RNA strands were amplified by PCR from DNA templates of LvRab11, 14-3-3ε and LacZ using specific primers (Supporting Table 1). Single-stranded RNAs (ssRNAs) were then produced in vitro using the RiboMAX Large-Scale RNA Production Systems SP6 and T7 (Promega) following the user's manual. To produce each dsRNA, sense and antisense strand of target dsRNAs were then added to same concentration in 1x annealing buffer II (100 mM Tris–HCl (pH 8.0), 10 mM EDTA and 1 mM NaCl). The annealing reaction was incubated at 70 °C for 10 min followed by slow cooling to room temperature. The quality of dsRNA was checked after annealing via gel electrophoresis. Then, shrimps size about 3–4 g were injected with 20 µg of each dsRNA (5 µg/g shrimp) dissolve in 100 µl of 1x PBS. Gills were collected from the shrimps 48 h after the dsRNA injection, then total RNA was extracted and assessed by RT-PCR using the corresponding primers (Supporting Table 1) to evaluate the efficacy of RNAi. To screen potential LvRab11 and 14-3-3ε with antiviral effects against WSSV, shrimps were divided into 4 groups: one control group receiving LacZ dsRNA injection and the other 3 RNAi groups receiving LvRab11 dsRNA, 14-3-3ε dsRNA, and both LvRab11 and 14-3-3ε dsRNA (LvRab11 + 14-3-3ε), respectively. At 48 h after the RNAi performance, each shrimp was challenged with 10^3^ copies of WSSV particles, and again 48 h later gill and pleopod tissues of shrimps were dissected and used to examine *ie1* mRNA expression and the virus copies by RT- qPCR with specific primers (Supporting Table 1).

### The *ie1* mRNA expression detection

Total RNA was extracted from gill tissue with TRIzol reagent (Invitrogen), and cDNA was synthesized as mention above. RT-qPCR was conducted to detect the mRNA levels of *ie1* under the RNAi experiments. RT-qPCR analysis was performed in the CFX Connect Real-Time PCR Detection System (Bio-Rad Laboratories) with a volume of 20 μl comprised of 200 ng cDNA, 10 μl of 2x SensiFAST SYBR No-ROX Mix (Bioline), and 400 nM of each primer (Supporting Table 1). The cycling programs were of the following parameters: 95 °C for 2 min to activate the polymerase, followed by 40 cycles of 95 °C for 5 s, 60 °C for 10 s, and 72 °C for 20 s. Expression level of each gene was calculated relative to the internal control gene *EF-1α* by using the the formula 2^−ΔΔCT^ method.

### Quantification of WSSV copy number

To evaluate the WSSV copy number, pleopods were used for DNA extraction. DNA was extracted using Genomic DNA isolation kit (Bio-Helix) following recommended protocols. Then, this DNA was used to evaluate the WSSV copy number using method as previously described^65^. Briefly, *VP28* gene was cloned into pGEM-T Easy vector (Promega). The quantification of purified recombinant plasmids were measured by NanoDrop 2000 spectrophotometer (Thermo Scientific) and its copy number was calculated. A ten-fold serially diluted solution of the plasmid DNA was used as standard sample to generate a standard curve in RT-qPCR, performed with VP28-F and VP28-R primers (Supporting Table 1) to determine the viral load. Each sample’s copy was converted to copies/ng DNA according to the DNA concentration measured above.

### Statistical analysis

The data are presented as the means ± standard deviation (SD) and were analysed using one-way ANOVA followed by an unpaired, two-tailed *t*-test. A measurement of *p* < 0.05 was accepted as statistically significant.

## Supplementary Information

Supplementary Information.
