# Supplementary material for: Evaluation of the relationship between the 14-3-3ε protein and LvRab11 in the shrimp Litopenaeus vannamei during WSSV infection
Source: Sci Rep. 2021 Sep 28;11:19188. doi: 10.1038/s41598-021-97828-w (PMC8478922; doi:10.1038/s41598-021-97828-w)
Supplement: Supplementary file 1 — Supplementary Information. [file 41598_2021_97828_MOESM1_ESM.pdf]

Evaluation of the relationship between the 14-3-3ε protein and LvRab11 in the shrimp *Litopenaeus vannamei* during WSSV infection

Guson Boonyoung[1], Tanate Panrat[3], Amornrat Phongdara[2], Warapond Wanna[1,2,*]

[1]Division of Biological Science, Faculty of Science, Prince of Songkla University, Hat Yai, Songkhla, Thailand, 90110

[2]Center for Genomics and Bioinformatics Research, Prince of Songkla University, Hat Yai, Songkhla, Thailand, 90110

[3]Prince of Songkla University International College, Prince of Songkla University, Hat Yai, Songkhla, Thailand, 90110

*Corresponding author. E-mail address: w.warapond@gmail.com

Present/permanent address: Division of Biological Science , Faculty of Science, Prince of Songkla University, Hat Yai, Songkhla, Thailand, 90110

Supporting data

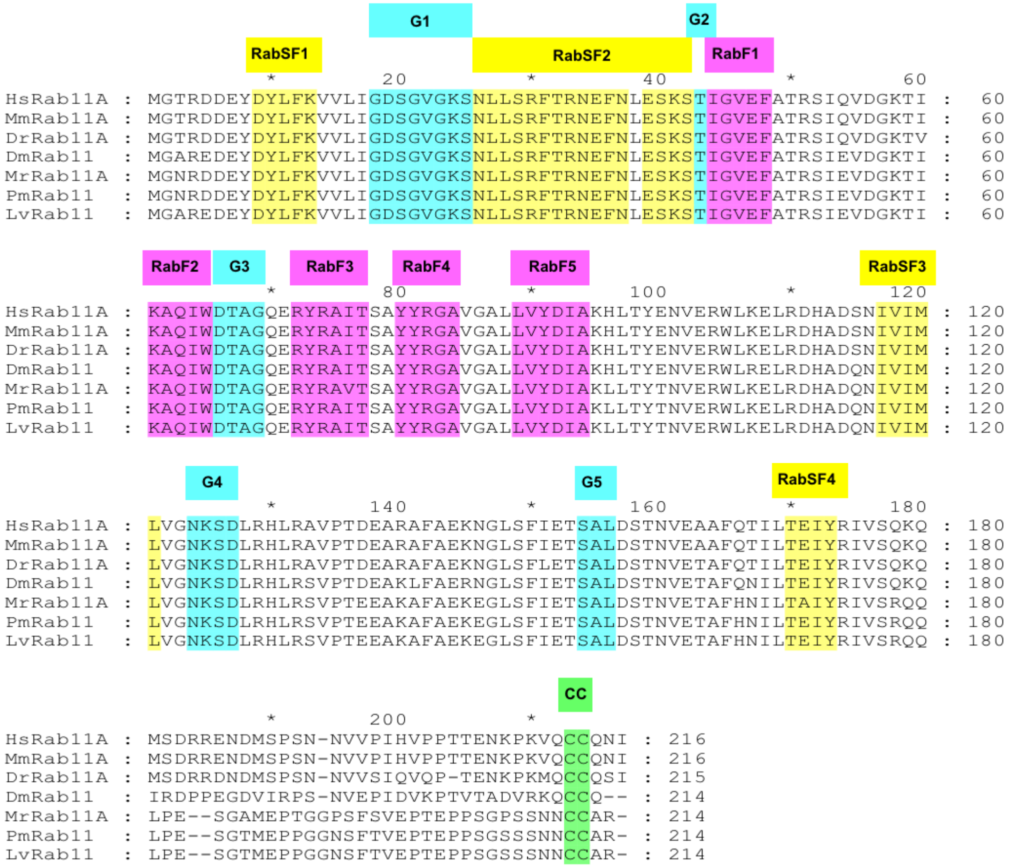

Supporting Figure 1. Multiple alignment of Rab11 in various species. The boxes indicate the characteristic motifs of the Rab family including GTP-binding site (G), Rab family-specific motif (RabF), Rab subfamily specific motif (RabSF), Cys–Cys motif (C motif). The GenBank accession numbers are as follows: HsRab11A, Rab11A of *H. sapiens* (CAG38732), MmRab11A , Rab11A of *M. musculus* (BAF02867), DrRab11A, Rab11A of *D. rerio* (NP_001007360), DmRab11, Rab11 of *D. melanogaster* (BAA21708), MrRab11A, Rab11A of *M. rosenbergii* (AJC97118), PmRab11, Rab11 of *P. monodon* (ASW35116.1), LvRab11, Rab11 of *L.vannamei*.

Ramachandran Plot
cj12

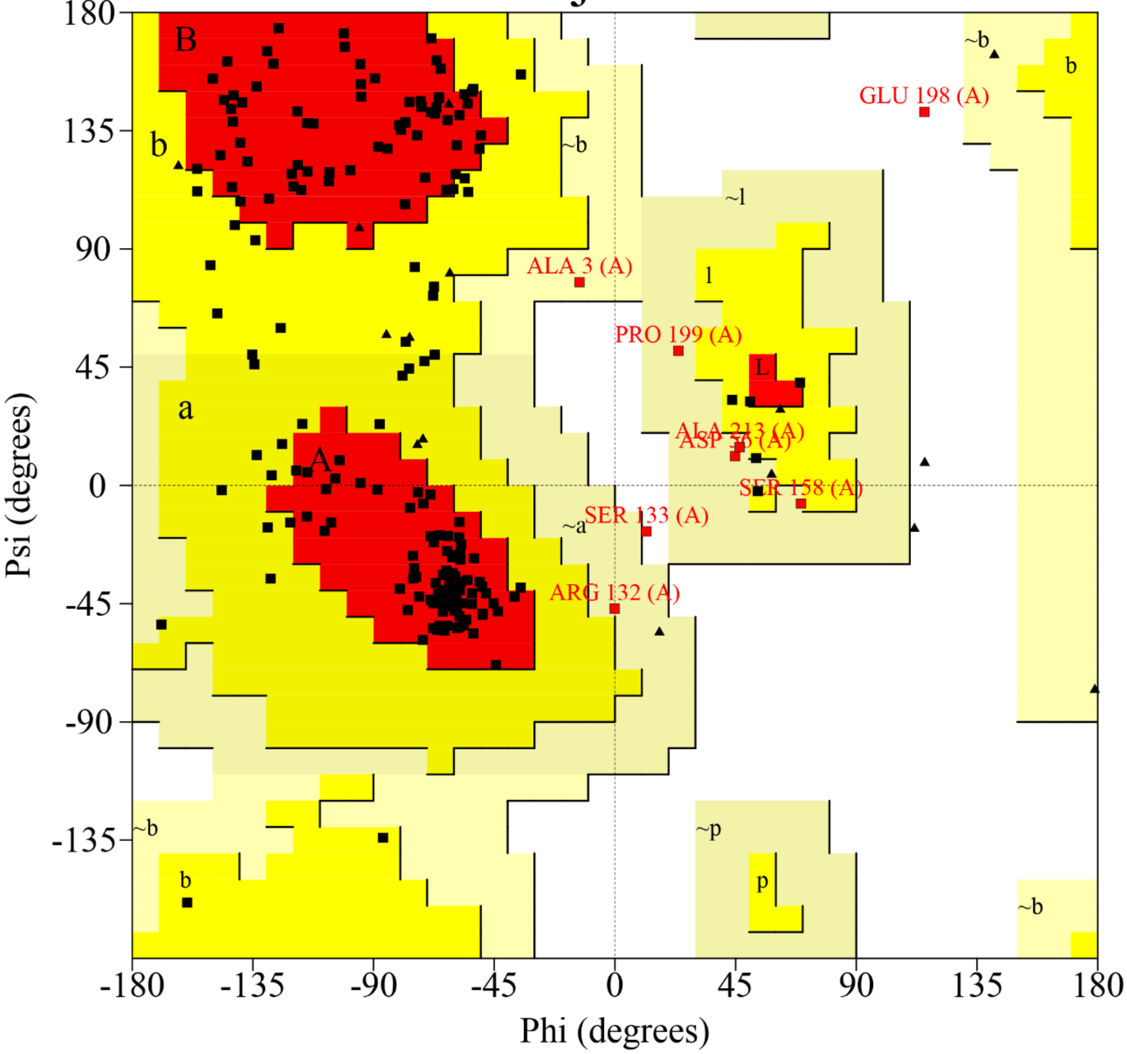

Plot statistics

Residues in most favoured regions [A,B,L]	149	78.4%
Residues in additional allowed regions [a,b,l,p]	34	17.9%
Residues in generously allowed regions [~a,~b,~l,~p]	5	2.6%
Residues in disallowed regions	2	1.1%
	----	------
Number of non-glycine and non-proline residues	190	100.0%
Number of end-residues (excl. Gly and Pro)	2	
Number of glycine residues (shown as triangles)	15	
Number of proline residues	7	

Total number of residues	214	

Supporting Figure 2. Ramachandran plot analysis of LvRab11 predicted structure. To assess the quality of the 3D predicted model, we applied the Ramachandran plot analysis on the ProFunc server (available at http://www.ebi.ac.uk/thornton-srv/databases/ProFunc). The analysis result of 214 amino acid residues of predicted structure found that 149 residues plotted in the most favored region, 34 residues in the additional allowed region, 5 residues in generously allowed regions, and 2 residues in the disallowed region. Additionally, 24 residues were found in Glycine, and Proline profile included the number of end-residues (Gly & Pro) was 2 residues, Glycine was 15 residues, and Proline was 7 residues, respectively.

Ramachandran Plot

cj13

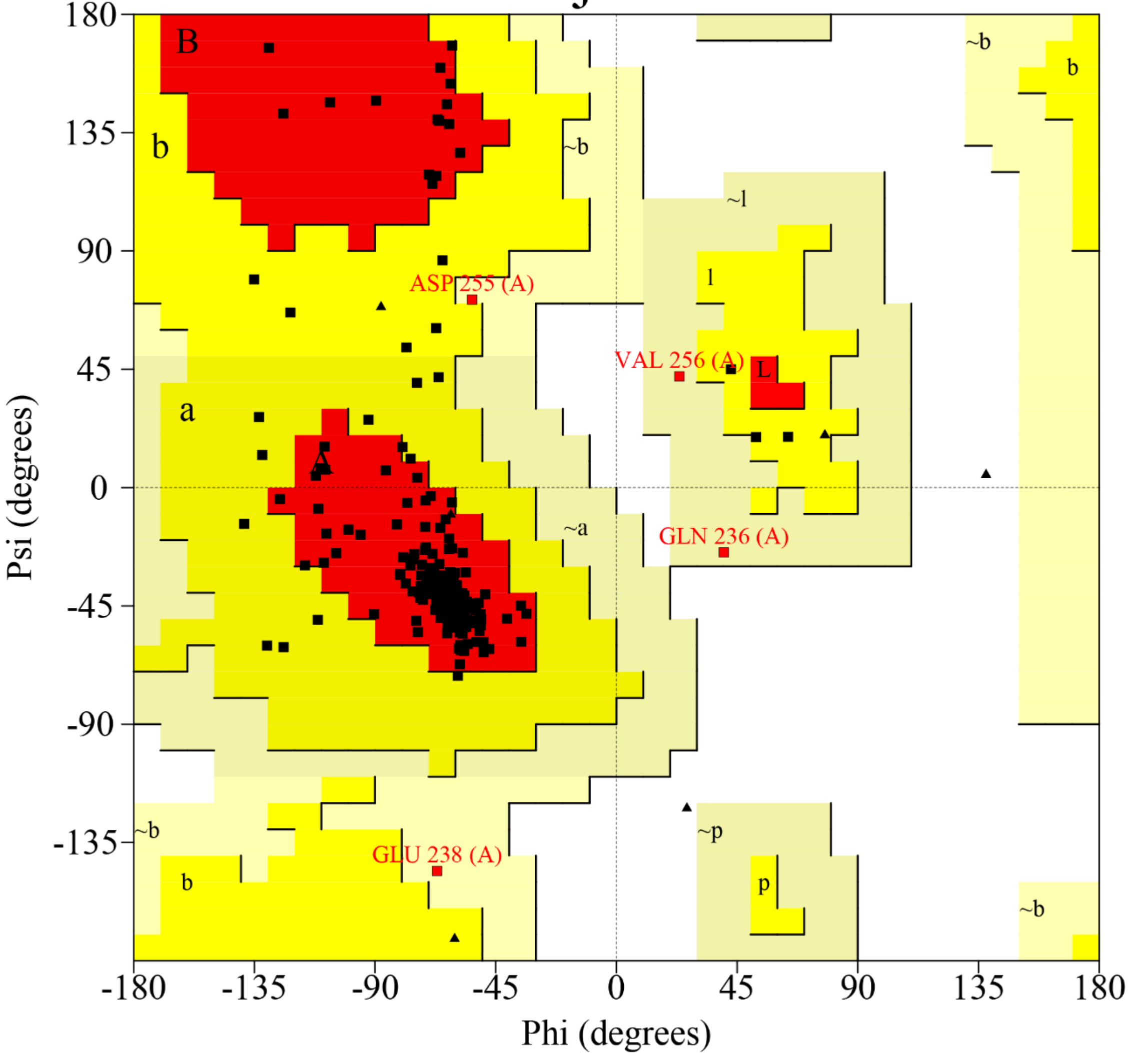

Plot statistics

Residues in most favoured regions [A,B,L]	215	90.0%
Residues in additional allowed regions [a,b,l,p]	20	8.4%
Residues in generously allowed regions [~a,~b,~l,~p]	4	1.7%
Residues in disallowed regions	0	0.0%
	----	------
Number of non-glycine and non-proline residues	239	100.0%
Number of end-residues (excl. Gly and Pro)	2	
Number of glycine residues (shown as triangles)	11	
Number of proline residues	5	

Total number of residues	257	

Supporting Figure 3. Ramachandran plot analysis of 14-3-3ES predicted structure. Ramachandran quality analysis result of 14-3-3ES predicted model showed 215 amino acid residues plotted in the most favored region, 20 residues in the additional allowed region and 4 residues in generously allowed regions. Additionally, 18 residues were found in Glycine, and Proline profile included the number of end-residues was 2 residues, Glycine was 11 residues, and Proline was 5 residues, respectively.

Ramachandran Plot

cj14

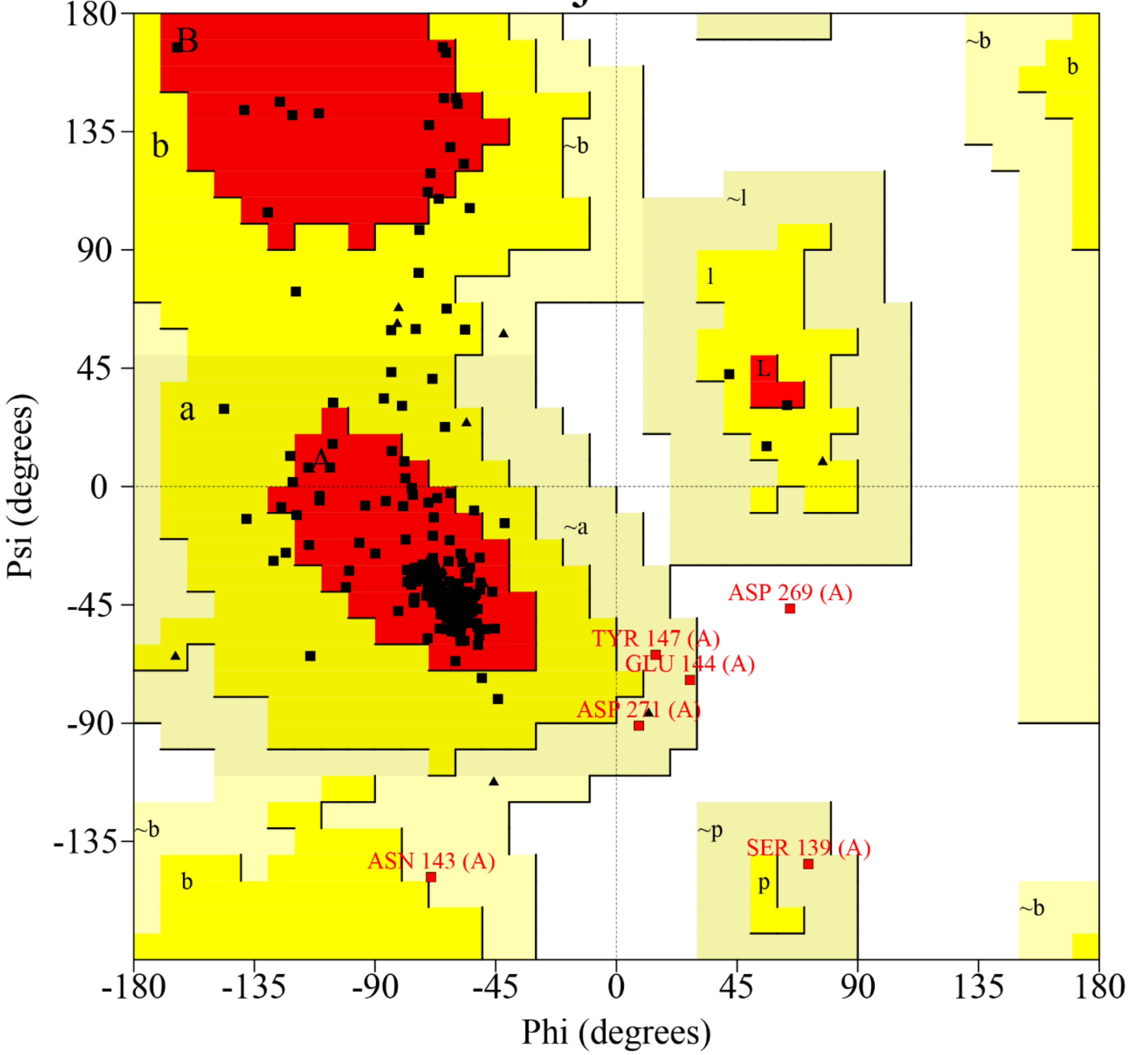

Plot statistics

Residues in most favoured regions [A,B,L]	219	86.6%
Residues in additional allowed regions [a,b,l,p]	28	11.1%
Residues in generously allowed regions [~a,~b,~l,~p]	5	2.0%
Residues in disallowed regions	1	0.4%
	----	------
Number of non-glycine and non-proline residues	253	100.0%
Number of end-residues (excl. Gly and Pro)	2	
Number of glycine residues (shown as triangles)	13	
Number of proline residues	5	

Total number of residues	273	

Supporting Figure 4. Ramachandran plot analysis of 14-3-3EL predicted structure. Ramachandran quality analysis result of 14-3-3EL predicted model showed 219 amino acid residues plotted in the most favored region, 28 residues in the additional allowed region, 5 residues in generously allowed regions, and one residue in the disallowed region. Additionally, 2 residues plotted in the end-residues, 13 residues as Glycine, and 5 as the Proline residues, respectively.

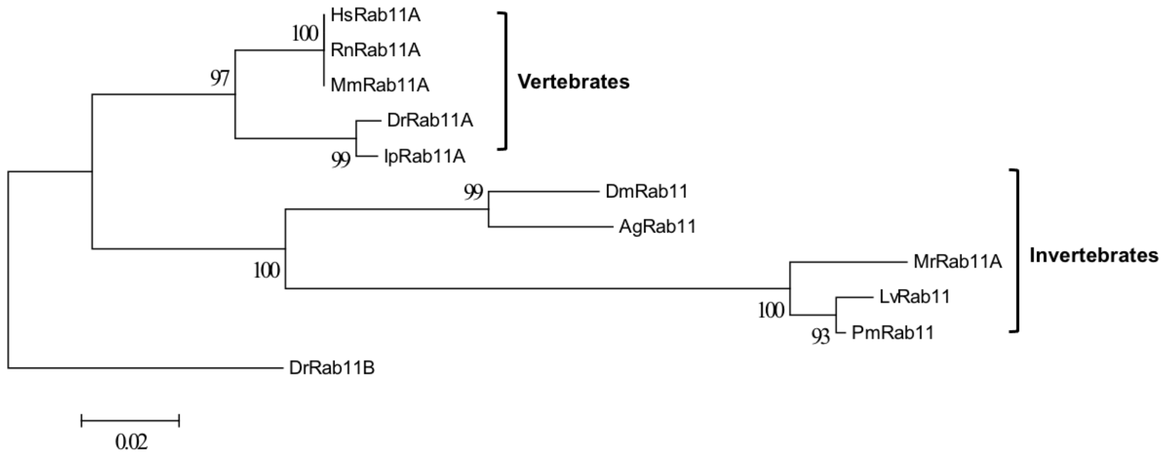

Supporting Figure 5. Phylogenetic tree of Rab11 protein in various organisms. The scale bar indicates amino acid substitutions per sites. The GenBank accession numbers are as follows: HsRab11A, Rab11A of *H. sapiens* (CAG38732), MmRab11A, Rab11A of *M. musculus* (BAF02867), RnRab11A, Rab11A of *Rattus norvegicus* (NP_112414), DrRab11A, Rab11A of *D. rerio* (NP_001007360), IpRab11A, Rab11A of *Ictalurus punctatus* (AHH37203), DrRab11B, Rab11B of *D. rerio* (NP_001002555), AgRab11, Rab11 of *Anopheles gambiae* (XP_001238825), DmRab11, Rab11 of *D. melanogaster* (BAA21708), MrRab11, Rab11 of *M. rosenbergii* (AJC97118), PmRab11, Rab11 of *P. monodon* (ASW35116.1), LvRab11, Rab11 of *L. vannamei*.

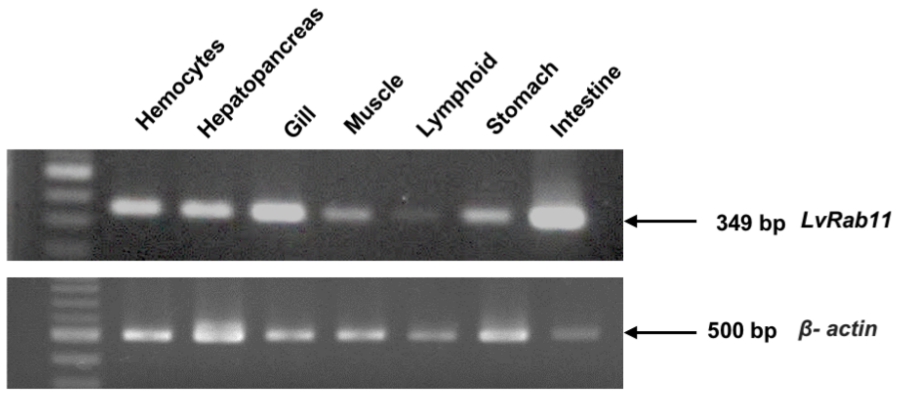

Supporting Figure 6. Tissue distributions of *LvRab11* in various tissues of shrimp detected by RT-PCR. *β-actin* gene was used as the internal reference. Tissues examined include hemocytes, hepatopancreas, gill, muscle, lymphoid, stomach and intestine. Expression levels of target genes were detected in three replicates for each tissue.

Primer name	Sequences (5'->3')	Experiments	Reference
Rab11-F	ATGGGBRCMMGVGAMGACGARTA	*LvRab11* amplification	This study
Rab11-R	TTSCCMACMAGCATRATRACRATGTT		
fullLvRab11-F	ATGGGTGCACGAGAAGACGAATA	Full length of *LvRab11* amplification and dsRNA construction	This study
fullLvRab11-R	TTAGCGGGCACAACAGTTGTTGGAG		
Rab11-RT-R	GTGTTAATTGGAGATTCGGGTGTTGG	RT-PCR and RT-qPCR	This study
Rab11-RT-F	GGTGACGCAAGTCAGATTTGTTACC		
Rab11-SmaI-F	CCCGGGGATGGGTGCACGAGA	pGADT7-LvRab11	This study
Rab11-XhoI-R	CTCGAGGCGGCACAACAGTTGTT		
Rab11-HindIII-pET-F	AAGCTTGGATGGGTGCACGAGAAGAC	pET-LvRab11	This study
Rab11-XhoI-pET-R	CTCGAGTTAGCGGGCACAACAGTT		
1433-F-NdeI	CATATGATGACGGACCGGGAGGACAAC	pGBKT7-1433ES<br>pGBKT7-1433EL	Wanna *et al.*[4]
1433-R-XhoI	CTCGAGTTAGCTCACATCCTGGTCCTCC		
1433-F-BamHI	GGATCCATGACGGACCGGGAGGACAAC	pET-1433ES<br>pET-1433EL	This study
1433-R-XhoI	CTCGAGTTAGCTCACATCCTGGTCCTCC		
1433-F-BamHI	GGATCCATGACGGACCGGGAGGACAAC	pGEX-1433ES<br>pGEX-1433EL	This study
1433-R-XhoI	CTCGAGTTAGCTCACATCCTGGTCCTCC		
14-3-3E-F	ATGACGGACCGGGAGGACAAC	dsRNA construction	This study
14-3-3E-R	TTAGCTCACATCCTGGTCCTCC		
14-3-3E2-F	TTACCACAGGTACCTCGCAGAA	RT-qPCR for 14-3-3ES	Wanna *et al.*[4]
14-3-3E-R	GCTGCATGATTAGGGTGGAGTC		
14-3-3E1wt48-F	CCGCCACTGGTAGCGAGAAGAA	RT-qPCR for 14-3-3EL	Wanna *et al.*[4]
14-3-3E-R	GCTGCATGATTAGGGTGGAGTC		
LacZ-F	ACCAGAAGCGGTGCCGGAAA	dsRNA construction	Sinthujaroen *et al.*[66]
LacZ-R	CCACAGCGGATGGTTCGGAT		
T7-R	TAATACGACTCACTATAGGG	dsRNA construction	Sinthujaroen *et al.*[66]
SP6-F	TATTTAGGTGACACTATAG		
VP28-F	GGATCTTTCTTTCACTCTTTC	WSSV copy number	This study
VP28-R	TCTGCCCCACAGTCACTTCGA		
ie1-Lo-F	TGGCACAACAACAGACCCTA	RT-qPCR for *ie*1	Huang *et al.*[67]
ie1-Lo-R	CTTTCCTTGCCGTACGAGAC		
β-actin-F	CAGATCATGTTYGAGACCTTC	RT-PCR and RT-qPCR for β-actin	Sinthujaroen *et al.*[66]
β-actin-R	GATGTCCACGTCRCACTTCAT		
EF1a-F	GAGCATACTGTTGGAAGGTCTCCA	RT-qPCR for *EF*-1α	This study
EF1a-R	GAACTGCTGACCAAGATCGACAGG		

Supporting Table 1. Primers used in this study.

Original images

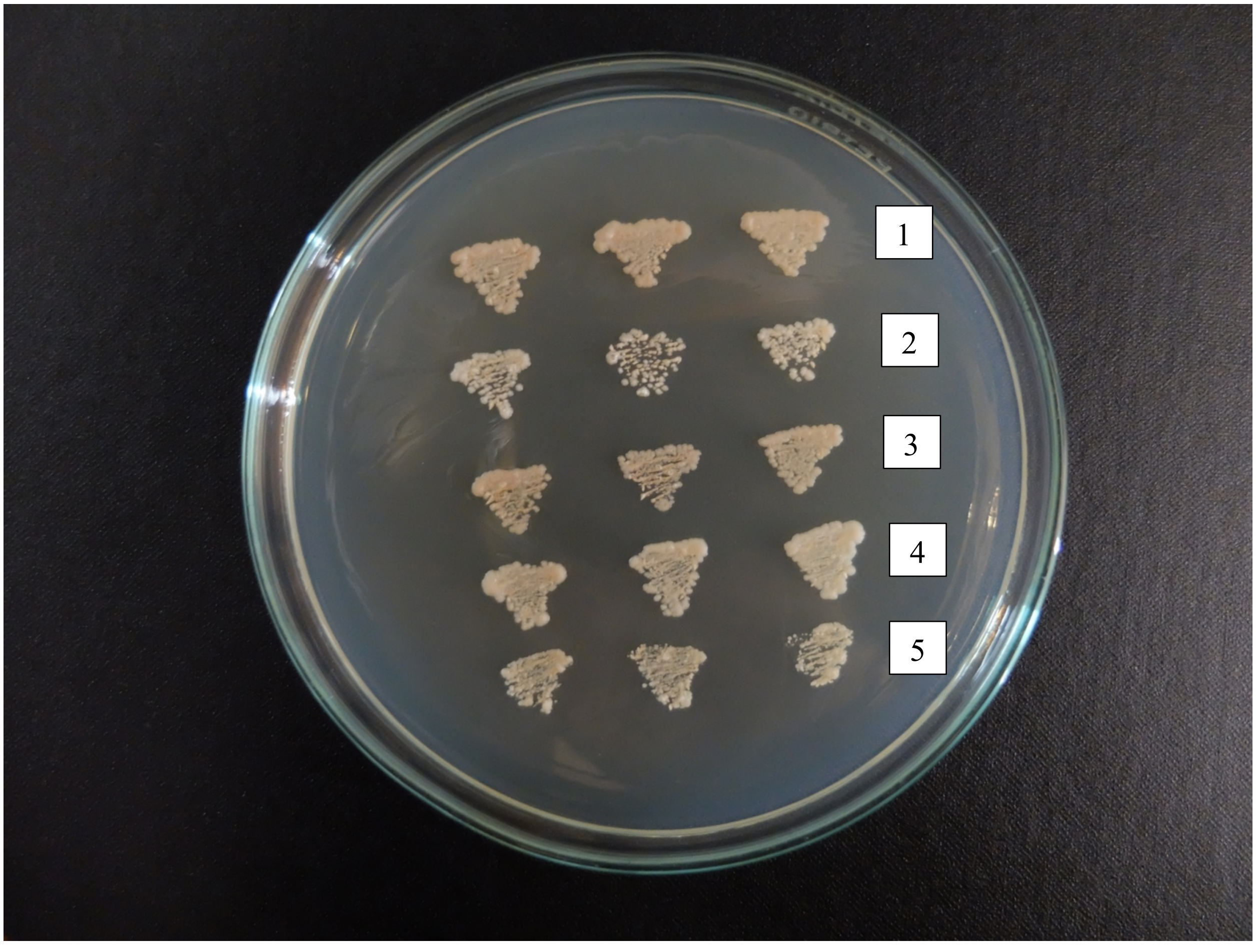

Figure 1A_original picture. Yeast (*Saccharomyces cerevisiae*) strain AH109 was co-transformed with the pGADT7 and pGBKT7 vector, which was divided into five groups: 1. pGADT7/pGBKT7 (negative control), 2. pGBKT7-53/pGADT7-T (positive control), 3. pGBKT7/ pGADT7-LvRab11, 4. pGBKT7-14-3-3EL/ pGADT7-LvRab11, 5. pGBKT7-14-3-3ES/ pGADT7-LvRab11. Yeast growth on solid SD/-Leu/-Trp at 30°C.

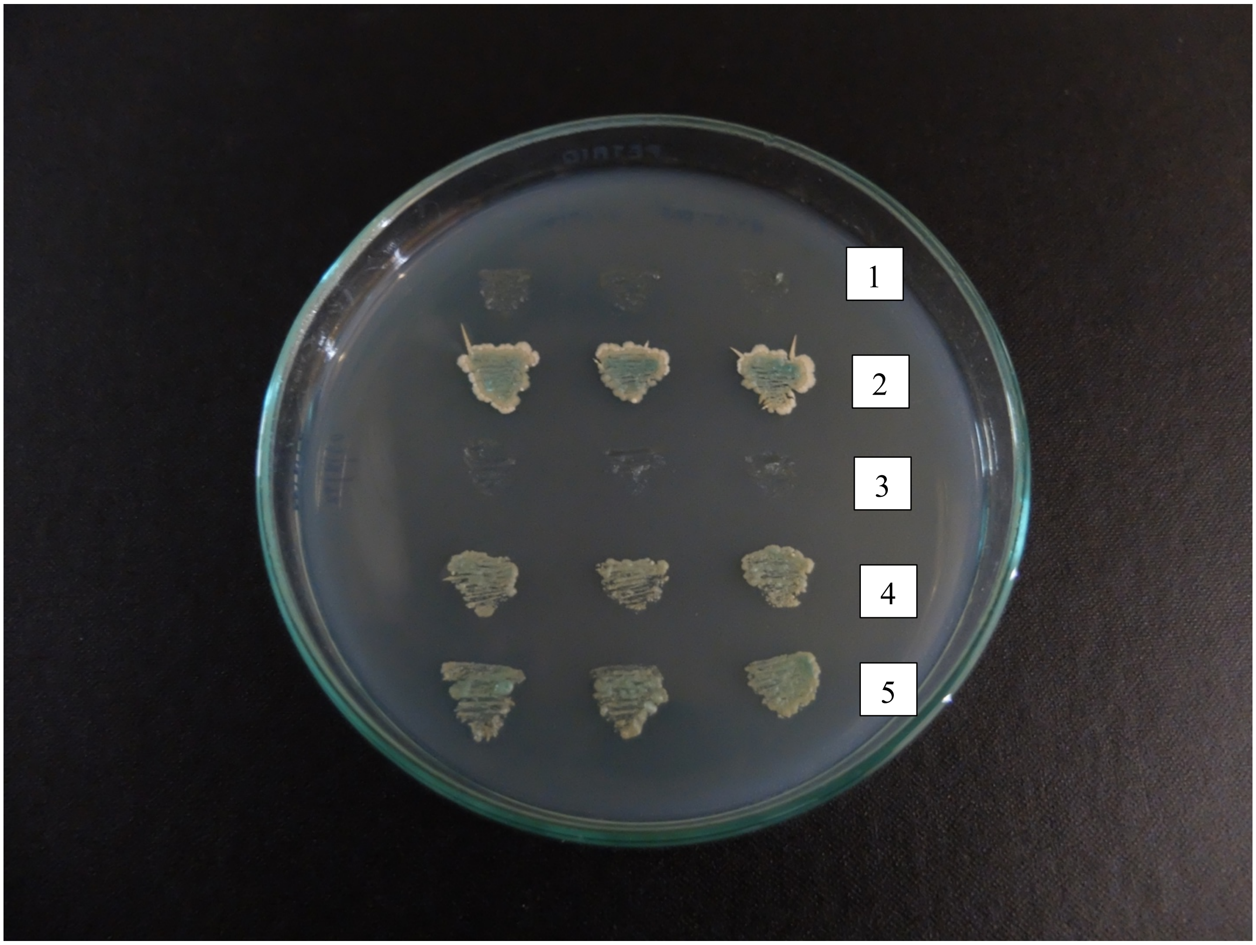

Figure 1A_original picture. Yeast (*Saccharomyces cerevisiae*) strain AH109 was co-transformed with the pGADT7 and pGBKT7 vector, which was divided into five groups: 1. pGADT7/pGBKT7 (negative control), 2. pGBKT7-53/pGADT7-T (positive control), 3. pGBKT7/ pGADT7-LvRab11, 4. pGBKT7-14-3-3EL/ pGADT7-LvRab11, 5. pGBKT7-14-3-3ES/ pGADT7-LvRab11. Yeast growth on solid SD/-Ade/-His-Leu/-Trp/X- α-gal at 30°C.

His-LvRab11	+	+	+	+
GST	-	+	-	-
GST-14-3-3EL	-	-	+	-
GST-14-3-3ES	-	-	-	+

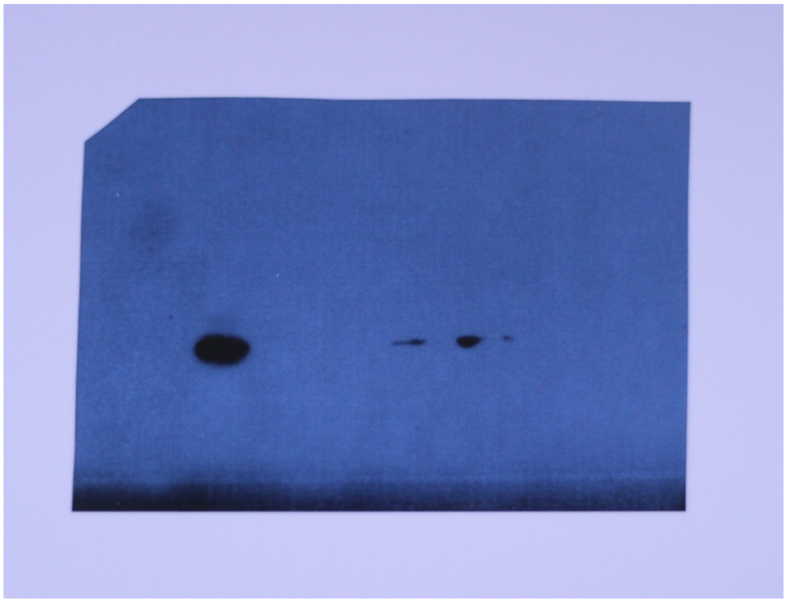

Figure 1B_original picture. Interaction was demonstrated by GST pull-down assays. GST pull-down assays using purified GST-14-3-3ES and GST-14-3-3EL recombinant proteins detected an interaction with His-LvRab11. The His-LvRab11 proteins in binding reactions were detected with goat-anti-mouse conjugated horseradish peroxidase bind with mouse anti-histidine antibodies.

His-LvRab11	+	+	+	+
GST	-	+	-	-
GST-14-3-3EL	-	-	+	-
GST-14-3-3ES	-	-	-	+

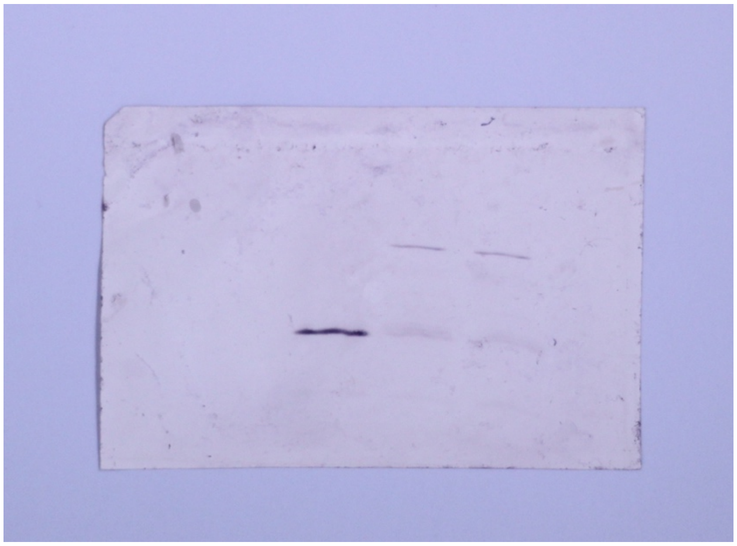

Figure 1B_original picture. Interaction was demonstrated by GST pull-down assays. GST pull-down assays using purified GST-14-3-3ES and GST-14-3-3EL recombinant proteins detected an interaction with His-LvRab11. The GST, GST 14-3-3EL and GST-14-3-3ES proteins in binding reactions were detected with rabbit-anti-goat conjugated alkaline phosphatase bind with goat-anti-GST antibodies.

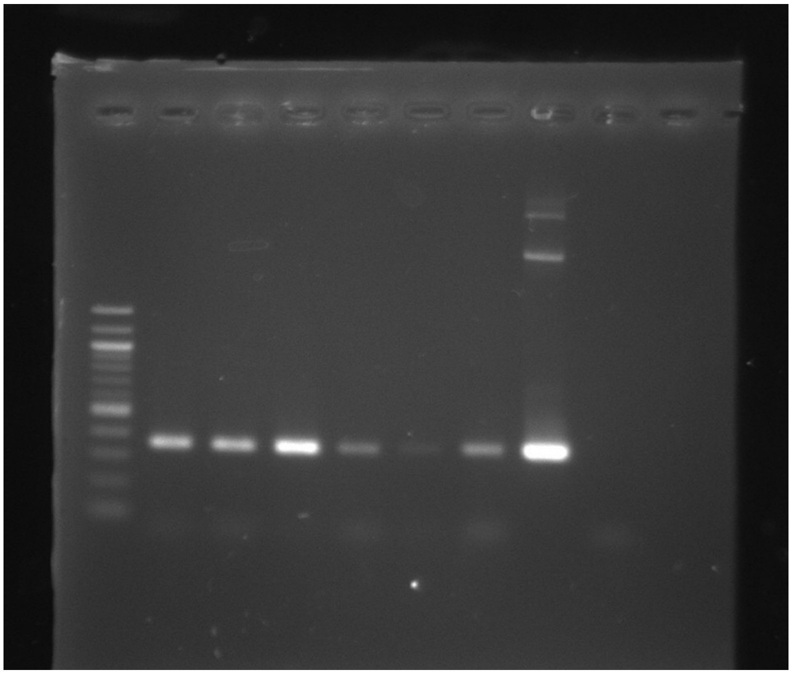

Supporting Fig 6_original picture. Tissue distributions of *LvRab11* in various tissues of shrimp detected by RT-PCR. Lane 1. 100 bp ladder, 2. hemocytes (He), 3. hepatopancreas (Hp), 4. gill (G), 5. muscle (M), 6. lymphoid (L), 7. stomach (S), 8. intestine (I) and 9. Negative control.

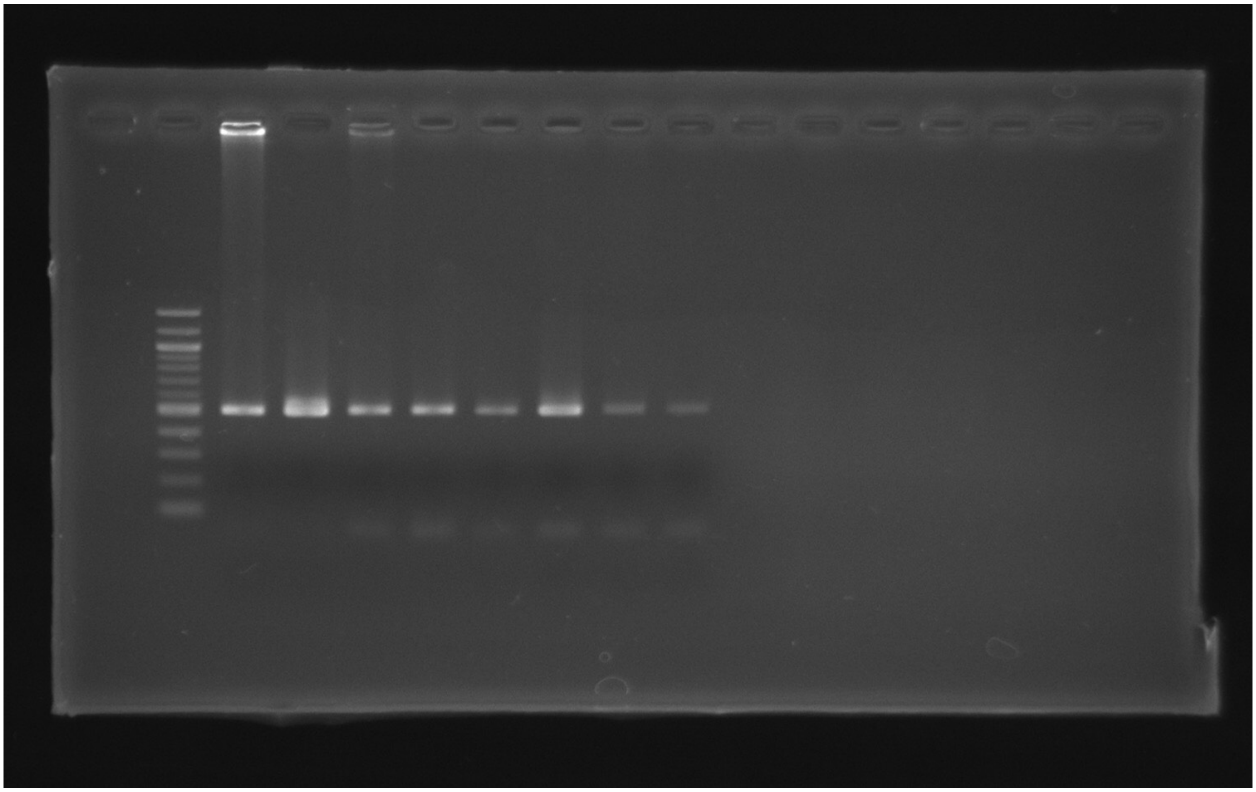

Supporting Fig 6_original picture. Tissue distributions of *β-actin* in various tissues of shrimp detected by RT-PCR. Lane 1. 100 bp ladder, 2. hemocytes (He), 3. hepatopancreas (Hp), 4. gill (G), 5. muscle (M), 6. lymphoid (L), 7. stomach (S) and 8,9. intestine (I).
